# Menopause-related quality of life 5 years after salpingectomy with delayed oophorectomy vs salpingo-oophorectomy

**DOI:** 10.1093/jnci/djag110

**Published:** 2026-04-07

**Authors:** Tamar A Gootzen, Rosella P M G Hermens, Marleen M H J van Gelder, Elle Heijnen, Majke H D van Bommel, Mirjam J A Apperloo, Monique M A Brood-van Zanten, Sjors F P J Coppus, Jose A E Custers, Helena C van Doorn, Katja N Gaarenstroom, Marline G Harmsen, Joanna IntHout, Marjan Knippenberg, Luc R C W van Lonkhuijzen, Marian J E Mourits, Jurgen M J Piek, Teska N Schuurman, Michiel Simons, Brigitte F M Slangen, Rachel Tros, M Caroline Vos, Ronald P Zweemer, C Marleen Kets, Joanne de Hullu, Miranda P Steenbeek

**Affiliations:** Department of Gynaecology and Obstetrics, Radboudumc, Nijmegen, The Netherlands; IQ Health, Radboudumc, Nijmegen, The Netherlands; IQ Health, Radboudumc, Nijmegen, The Netherlands; Department of Gynaecology and Obstetrics, Radboudumc, Nijmegen, The Netherlands; Department of Gynaecology and Obstetrics, Radboudumc, Nijmegen, The Netherlands; Department of Obstetrics and Gynecology, Frisius MC, Leeuwarden, The Netherlands; Department of Gynecology, Netherlands Cancer Institute/Antoni van Leeuwenhoek, Amsterdam, The Netherlands; Department of Obstetrics and Gynecology, Maxima Medical Centre, Veldhoven, The Netherlands; Department of Medical Psychology, Radboudumc, Nijmegen, The Netherlands; Department of Gynecology, Erasmus MC Cancer Clinic, Rotterdam, The Netherlands; Department of Obstetrics and Gynecology, Leiden University Medical Centre, Leiden, The Netherlands; Department of Gynaecology and Obstetrics, Radboudumc, Nijmegen, The Netherlands; IQ Health, Radboudumc, Nijmegen, The Netherlands; IQ Health, Radboudumc, Nijmegen, The Netherlands; Center for Gynecologic Oncology, Cancer Center Amsterdam UMC, Amsterdam, The Netherlands; Department of Obstetrics and Gynecology, University Medical Center Groningen, University of Groningen, Groningen, The Netherlands; Department of Gynaecology and Obstetrics and Catharina Cancer Institute, Catharina Hospital, Eindhoven, The Netherlands; Department of Gynaecology, Netherlands Cancer Institute, Antoni van Leeuwenhoek, Amsterdam, The Netherlands; Department of Pathology, Radboudumc, Nijmegen, The Netherlands; Department of Obstetrics and Gynecology, Maastricht University Medical Centre, GROW-School for Oncology and Developmental Biology, Maastricht, The Netherlands; Center for Gynecologic Oncology, Cancer Center Amsterdam UMC, Amsterdam, The Netherlands; Department of Obstetrics and Gynaecology, Elisabeth-TweeSteden Hospital, Tilburg, The Netherlands; Department of Gynecological Oncology, UMC Utrecht, Utrecht, The Netherlands; Department of Human Genetics, Radboudumc, Nijmegen, The Netherlands; Department of Gynaecology and Obstetrics, Radboudumc, Nijmegen, The Netherlands; Department of Gynaecology and Obstetrics, Radboudumc, Nijmegen, The Netherlands

## Abstract

**Background:**

*BRCA1/2* pathogenic variant (PV) carriers have an increased risk of tubo-ovarian cancer. They are recommended to have a risk-reducing salpingo-oophorectomy around age 40, resulting in premature menopause. The alternative risk-reducing salpingectomy (RRS) with delayed oophorectomy (DO) postpones this. We present the 5-year results of a nationwide preference trial (TUBA study) comparing menopause-related quality of life (QoL) after RRS/DO with salpingo-oophorectomy.

**Methods:**

Premenopausal *BRCA1/2*-PV carriers aged 25-40 (*BRCA1*) or 25-45 (*BRCA2*) chose RRS/DO or salpingo-oophorectomy with or without hormonal replacement therapy (HRT). We compared QoL (Greene Climacteric Scale) between RRS/DO and salpingo-oophorectomy without HRT 5 years after surgery using linear mixed models. Secondarily, RRS/DO was compared with salpingo-oophorectomy with HRT. Women who underwent oophorectomy in the RRS/DO group were excluded in a sensitivity analysis.

**Results:**

In total, 410 (71.9%) participants chose RRS/DO and 160 (28.1%) salpingo-oophorectomy. Seventy-three (17.8%) participants underwent oophorectomy within 5 years after salpingectomy. The adjusted mean difference (aMD) in QoL was 1.8 (95% CI = −0.6 to 4.3) after salpingo-oophorectomy without HRT and 1.4 (95% CI = −0.4 to 3.1) after salpingo-oophorectomy with HRT compared with RRS (with and without oophorectomy) at 5 years follow-up. The sensitivity analysis showed that QoL decreased less after RRS before oophorectomy compared with salpingo-oophorectomy without (aMD = 2.4, 95% CI = 0.1 to 4.8) and with HRT (aMD = 1.9, 95% CI = 0.1 to 3.7).

**Conclusion:**

Postponing oophorectomy is key in preventing deterioration of QoL. QoL was similar 5 years after salpingectomy (with and without oophorectomy) compared with salpingo-oophorectomy (regardless of HRT use). However, QoL was higher when comparing salpingectomy without oophorectomy to salpingo-oophorectomy (regardless of HRT use).

## Introduction

Tubo-ovarian cancer is the gynaecologic malignancy with the highest mortality rate.^1^ Women carrying a *BRCA1/2* pathogenic variant (PV) have an increased lifetime risk of tubo-ovarian cancer (39%-58% *BRCA1*; 13%-29% *BRCA2*).^2^^,^^3^ As no effective screening methods are currently available.^4^^,^^5^  *BRCA1/2*-PV carriers are advised to undergo a risk-reducing salpingo-oophorectomy (RRSO) (age 35-40 *BRCA1*; 40-45 *BRCA2*).^6^

RRSO reduces tubo-ovarian cancer risk by 96%.^7^ It results in premature menopause, which is associated with negative short-term (eg, hot flushes, sleep disturbances, and impaired sexual functioning)^8^ and long-term (increased risk of cardiovascular diseases, cognitive impairment, and osteoporosis) effects.^9^ To alleviate menopausal symptoms after salpingo-oophorectomy, hormone replacement therapy (HRT) until the age of natural menopause is recommended unless contraindicated, for example, in women with a history of breast cancer or thrombosis.^10^^,^^11^

Growing evidence indicates that high-grade serous carcinomas originate in the fallopian tubes rather than the ovaries.^12^^,^^13^ This resulted in an alternative preventive strategy in *BRCA1/2*-PV carriers: a risk-reducing salpingectomy (RRS) with delayed oophorectomy (DO). The major advantage of this strategy is that iatrogenic menopause can be postponed.^14^ The only consequence is that natural conception is no longer possible after salpingectomy. Therefore, choosing RRS with DO potentially leads to earlier risk reduction.

In previous short-term analyses of the TUBA study, RRS/DO was compared with the standard salpingo-oophorectomy in *BRCA1/2*-PV carriers regarding quality of life (QoL).^15^ Until 3 years post-surgery, *BRCA1/2*-PV carriers who opted for salpingo-oophorectomy (with or without HRT) had a significantly larger decline in menopause-related QoL and sexual functioning than those who chose RRS/DO.^16^^,^^17^ These differences increased in the first year after surgery but became less pronounced 3 years post-surgery. However, the long-term effects of RRS/DO and specifically the impact of the DO on QoL are still unknown.

Therefore, this article aims to evaluate the difference in menopause-related QoL between RRS/DO and RRSO in *BRCA1/2-*PV carriers 5 years after first surgery.

## Methods

### Study design and population

The study design of this Dutch preference trial was previously published.^15^  *BRCA1/2-*PV carriers chose either the alternative RRS/DO or the standard RRSO with or without HRT afterward. The salpingectomy could be performed between the ages of 25-40 (*BRCA1*) or 25-45 (*BRCA2*) and the oophorectomy up to the maximum age of 45 (*BRCA1*) or 50 (*BRCA2*). The salpingo-oophorectomy could be executed between the guideline ages of 35-40 (*BRCA1*) or 40-45 (*BRCA2*).^6^ Inclusion criteria consisted of women who (1) were premenopausal, (2) had a *BRCA1/2*-PV, (3) were aged 25-40 (*BRCA1*) or 25-45 (*BRCA2*), (4) completed childbearing, and (5) had at least 1 fallopian tube present. Exclusion criteria included (1) a history of tubo-ovarian, fallopian tube, or peritoneal cancer; (2) current diagnosis or current treatment of a malignant disease; (3) legal incapability; and (4) inability to read or speak Dutch. Participants were recruited from 13 Dutch hospitals between January 2015 and November 2019.

Use of HRT was recommended after removal of the ovaries, if not contraindicated.^11^ Participants completed web-based questionnaires at baseline, 3 months, and 1 year after first surgery, and biennially thereafter up to 15 years post-surgery.

### Safety

The TUBA study was approved by the Medical Ethics Committee of Arnhem-Nijmegen; an independent, multidisciplinary data safety monitoring board was installed; and a safety rule was established. This entailed monitoring of tubo-ovarian cancers in the RRS/DO group and an alert when this exceeded the expected incidence. Until July 2025, no interval tubo-ovarian cancer cases were observed among participants with normal pathology at (first) risk-reducing surgery in all groups.

### Outcomes

Usage of HRT (in women without a prior history of breast cancer), type of HRT, and uptake and timing of oophorectomy were described. The primary outcome was menopause-related QoL, assessed with the validated Greene Climacteric Scale (GCS).^18^ It consisted of 21 menopause-related symptoms in the subdomains anxiety, depression, somatic, vasomotor, and sexual problems. The score ranges from 0 to 63, with a higher score indicating more climacteric symptoms. A 2-point difference in GCS indicates mild to moderate worsening in 2 climacteric symptoms. Secondary outcomes included (1) sexual functioning, assessed with the Female Sexual Functioning Index (FSFI);^19^ (2) sexual distress, assessed with the Female Sexual Distress Scale (FSDS);^20^ (3) cancer worry, assessed with the Cancer Worry Scale;^21^ (4) decisional regret, assessed with the Decisional Regret Scale (DRS);^22^ and (5) health-related QoL, assessed with the Short Form Health Survey 36 (SF-36).^23^ Outcomes were compared between three groups: RRS/DO, RRSO without HRT, and RRSO with HRT. Last, the secondary outcomes surgical and histopathological outcomes were described.

### Statistical analysis

Baseline characteristics were described using descriptive statistics. Baseline HRT use was assessed at 3 months, or if 3-month follow-up data were missing, at 1 year. HRT use during follow-up was based on self-reported use in each questionnaire. Participants could start or discontinue HRT during the follow-up period, resulting in participants switching between the “RRSO with HRT” and “RRSO without HRT” groups across different follow-up points (eg, when a participant initiated HRT after RRSO but discontinued 2 years after RRSO she is in the RRSO with HRT group at 3-months and 1-year follow-up and in the RRSO without HRT group at 3- and 5-year follow-up).

The primary outcome was assessed using the adjusted mean difference (aMD) in GCS score (difference between baseline and follow-up moment) with a linear mixed model. We corrected for differences at baseline by using the aMD. We adjusted for baseline GCS score and baseline SF-36 mental and physical component scores^23^ as well as two 3-way interactions: (1) type of strategy, follow-up time point, and age at first surgery, and (2) type of strategy, follow-up time point, and baseline GCS score. All corresponding 2-way interactions were incorporated. Type of *BRCA1/2*-PV (*BRCA1* vs *BRCA2*) and age at first surgery were added based on clinical relevance. The same applied to the interaction between type of strategy and follow-up time point (3 months, 1 year, 3 years, and 5 years post-surgery), as the trajectory of menopause-related QoL over time is not constant. Furthermore, a random effect for center of inclusion was added to account for clustering of patients within centers and an unstructured correlation matrix for the within-participants correlation between repeated measurements (3 months, 1 year, 3 years, and 5 years post-surgery) was added to account for dependence within the different measurements for 1 participant.

RRS/DO was compared with RRSO without HRT and RRSO with HRT for all secondary outcomes. For each outcome, a separate linear mixed model was fitted, using the same structure of fixed and random effects as for the primary outcome. Each model was adjusted for the baseline value of the corresponding outcome variable, and fixed effects were reassessed for statistical significance to account for potential outcome-specific variation. To assess the timing of oophorectomy execution, descriptive statistics were used.

We performed 2 sensitivity analyses to assess robustness of the findings. In the first sensitivity analysis, participants in the RRS/DO group were excluded from the analysis from the moment of their oophorectomy. This allowed us to evaluate the impact of RRS without the subsequent oophorectomy. The second analysis excluded participants who underwent their first surgery before the recommended RRSO guideline age (<35 years, *BRCA1*; <40 years, *BRCA2*). We aimed to minimize the impact of absolute age, as women choosing for RRS/DO could have their salpingectomy before the guideline age, whereas women opting for RRSO could not. Both analyses were conducted using the previously described model structure. If the sensitivity analysis showed a difference compared with the primary outcome, the sensitivity analysis was performed for all secondary outcomes. Data were analyzed using SPSS version 29 (IBM Corp).

## Results

### Study population

**Figure 1.**
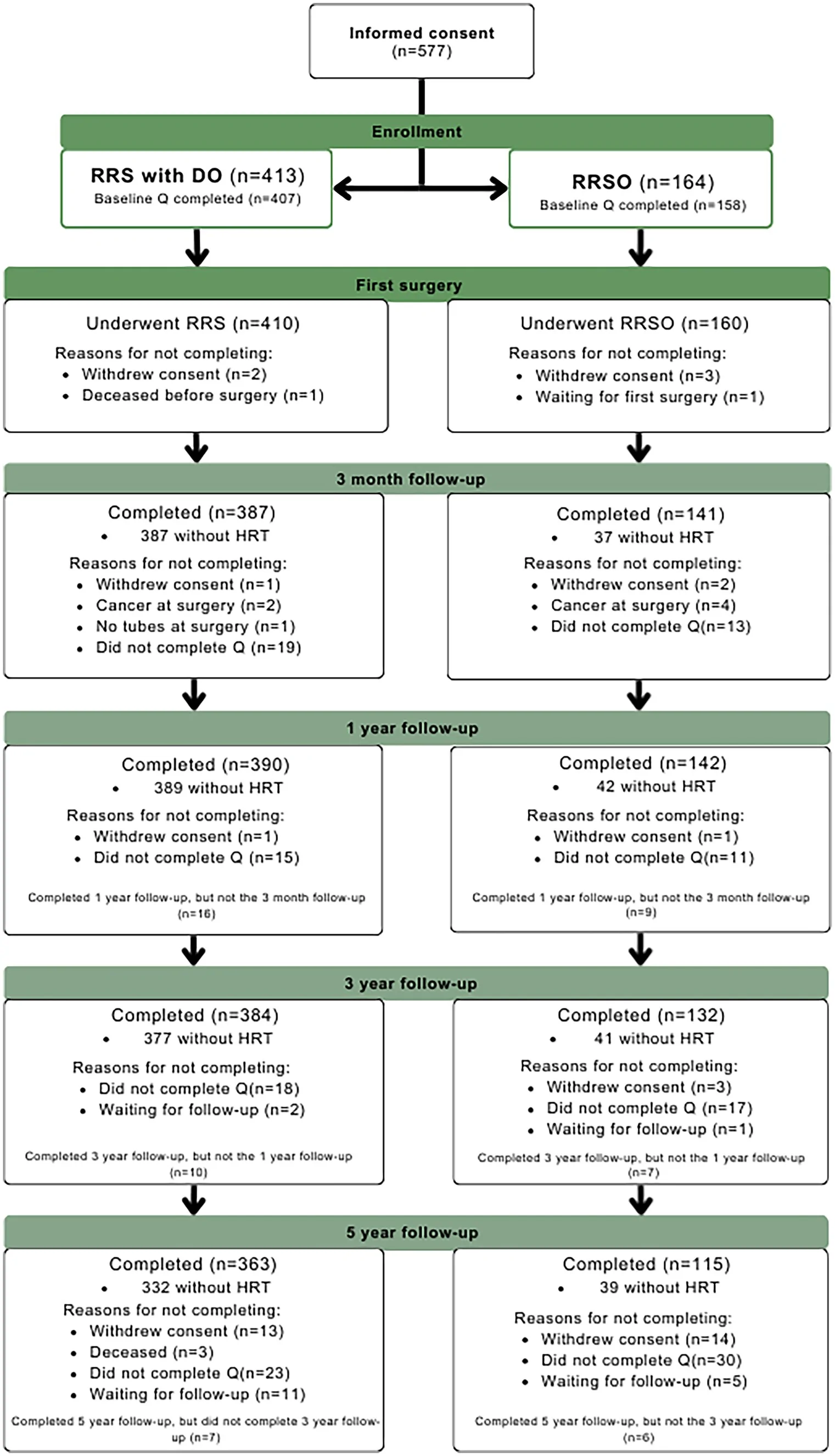
Flowchart of participants. Abbreviations: DO = delayed oophorectomy; HRT = hormone replacement therapy; Q = questionnaire; RRS = risk-reducing salpingectomy; RRSO = risk-reducing salpingo-oophorectomy.

In total, 577 participants provided informed consent between January 2015 and November 2019 (Figure 1). Our analysis was performed on October 8, 2025. A total of 410 (71.1%) underwent RRS/DO, and 160 (27.7%) underwent salpingo-oophorectomy. Five-year follow-up questionnaires were completed by 363 (88.5%) and 115 (71.8%) participants, respectively. Participants in the RRS/DO group (median age = 38, interquartile range [IQR] = 35-40) were younger than participants in the RRSO without HRT (39, IQR = 37-39) and with HRT (40, IQR = 36-39) groups at first surgery. Personal history of breast cancer (at baseline) was highest in the RRSO without HRT group (52.0%), followed by the RRS/DO group (14.6%) and lowest in the RRSO with HRT group (0.9%) (Table 1).

**Table 1.** Baseline characteristics per group and stratified by HRT use at baseline.^a^

	RRS/DO		RRSO without HRT		RRSO with HRT	
	N/Median	%/IQR	N/Median	%/IQR	N/Median	%/IQR
Participants who underwent first surgery	N = 410		N = 50		N = 110	
Type of *BRCA* PV						
*BRCA1*	196	47.8%	30	60.0%	67	60.9%
*BRCA2*	214	52.2%	20	40.0%	43	39.1%
Age at first surgery, y	38	35-40	40	37-32	39	36-31
*BRCA1*	36	34-38	37	36-39	38	36-39
*BRCA2*	39	37-41	41	40-44	42	41-43
Time between *BRCA* diagnosis and surgery						
<5 years	192	46.8%	25	50.0%	56	50.9%
≥5 years	213	52.0%	22	44.0%	52	47.3%
Unknown	5	1.2%	3	6.0%	2	1.8%
Relationship status						
Married/cohabitating	363	88.5%	42	84.0%	101	91.8%
Single	42	10.2%	5	10.0%	8	7.3%
Unknown	5	1.2%	3	6.0%	1	0.9%
Offspring						
No	49	12.0%	9	18.0%	7	6.4%
Yes	355	86.6%	37	74.0%	101	91.8%
Unknown	6	1.5%	4	8.0%	2	1.8%
Level of education						
Low	46	11.2%	7	14.0%	12	10.1%
Medium	143	34.9%	18	36.0%	39	35.5%
High	216	52.7%	22	44.0%	58	52.7%
Unknown	5	1.2%	3	6.0%	1	0.9%
Breast cancer in personal history						
No	345	84.1%	26	52.0%	108	98.2%
Yes	60	14.6%	21	42.0%	1	0.9%
Unknown	5	1.2%	3	6.0%	1	0.9%
Timing of risk-reducing mastectomy						
Prior to first surgery	160	39.0%	20	40.0%	37	33.6%
During first surgery	1	0.2%	1	2.0%	0	NA
Timing unknown	1	0.2%	0	NA	1	0.9%
No risk-reducing mastectomy^b^	243	59.3%	26	52.0%	71	64.5%
Unknown	5	1.2%	3	6.0%	1	0.9%
First-degree relative with breast cancer						
No	205	50.0%	25	50.0%	64	58.2%
Yes	199	48.5%	22	44.0%	44	40.0%
Unknown	6	1.5%	3	6.0%	2	1.8%
First-degree relative with ovarian cancer						
No	354	86.3%	41	82.0%	87	79.1%
Yes	50	12.2%	6	12.0%	21	25.5%
Unknown	6	1.5%	3	6.0%	2	1.8%

a Baseline HRT use was determined using the 3-month questionnaire, supplemented with data from the 1-year questionnaire for those who did not complete the 3-month questionnaire.

b Prior to first surgery.

Abbreviations: DO = delayed oophorectomy; N = number of participants; NA = not applicable; HRT = hormone replacement therapy; RRS = risk-reducing salpingectomy; RRSO = risk-reducing salpingo-oophorectomy.

### Timing of DO

**Figure 2.**
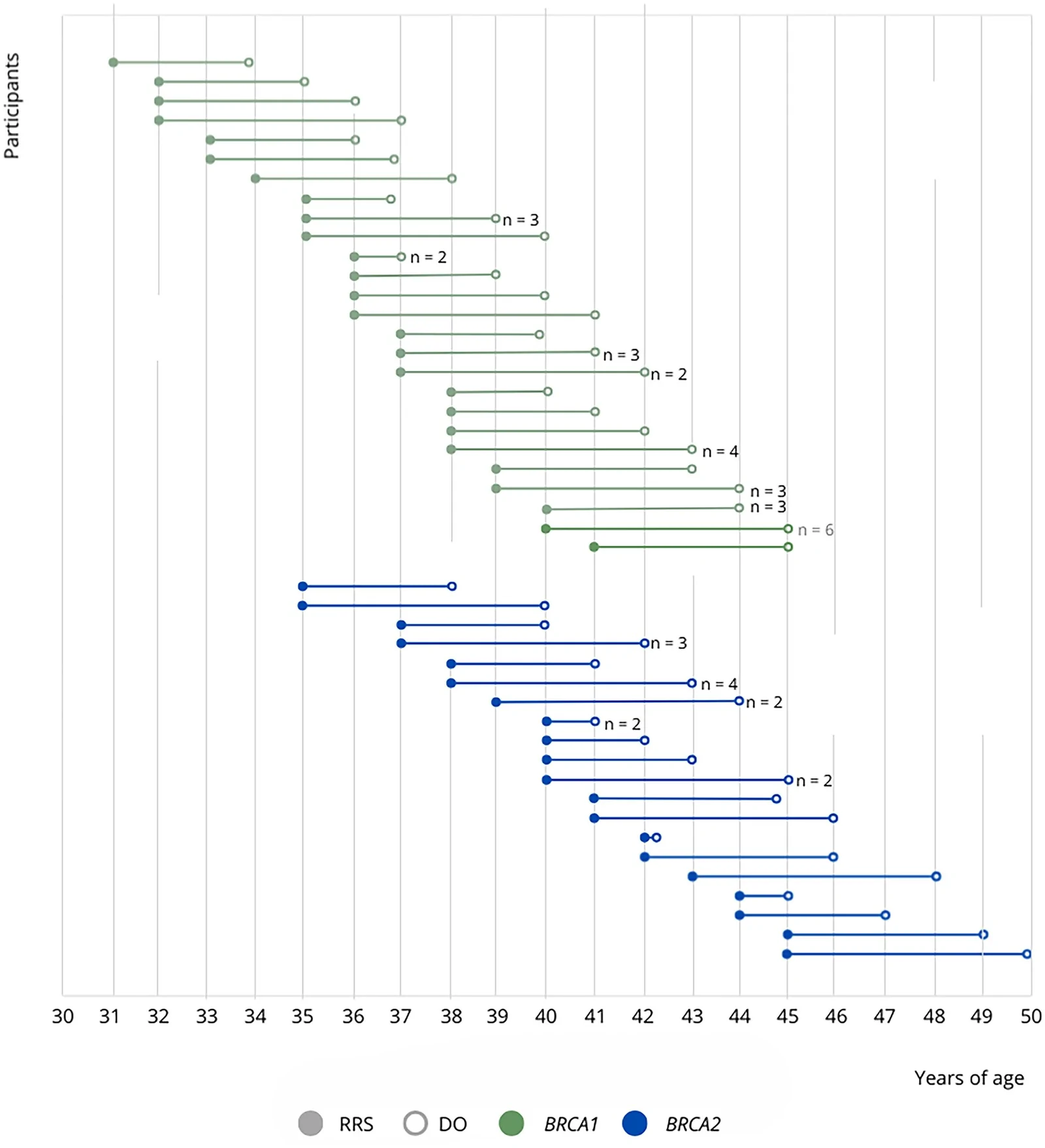
Timing of RRS with DO. Each horizontal line represents a participant who underwent her RRS at the age indicated by the first (**solid**) dot and her DO at the age indicated by the second (**open**) dot. Abbreviations: DO = delayed oophorectomy; RRS = risk-reducing salpingectomy.

A total of 17.6% (72/410) participants underwent their oophorectomy within 5 years after salpingectomy (Figure 2). Median (IQR) time between salpingectomy and oophorectomy was 4 (3; 5) years in *BRCA1*- and *BRCA2*-PV carriers. A total of 56/72 (77.8%) had their DO within the recommended RRSO guideline age range. Participants underwent their oophorectomy at a median age of 42 (39; 42) years (*BRCA1*) and 43 (42; 46) years (*BRCA2*). Seven *BRCA1*-PV carriers (15.9%) postponed their oophorectomy until age 45, the recommended upper limit in the study. Of the *BRCA2*-PV carriers, 1 carrier (3.5%) had her oophorectomy at age 50.

### HRT use

HRT use after salpingo-oophorectomy in women without a prior history of breast cancer slightly decreased over time from 85.6% (3 months), to 82.4% (1 year), 83.3% (3 years), and 82.7% (5 years). A total of 72 participants had their DO within 5 years after salpingectomy, of which 20 (27.8%) had breast cancer before RRO. In total 34/52 (65.4%) participants without a prior history of breast cancer started HRT after RRS/DO. Tibolone was the predominant type of HRT at 3-month follow-up. Over the years, use of tibolone gradually declined, as it was increasingly replaced by other types of HRT, such as estradiol patches, gel, or spray in combination with progesterone capsules or a levonorgestrel intra-uterine device (Table S1).

### Primary outcome

**Figure 3.**
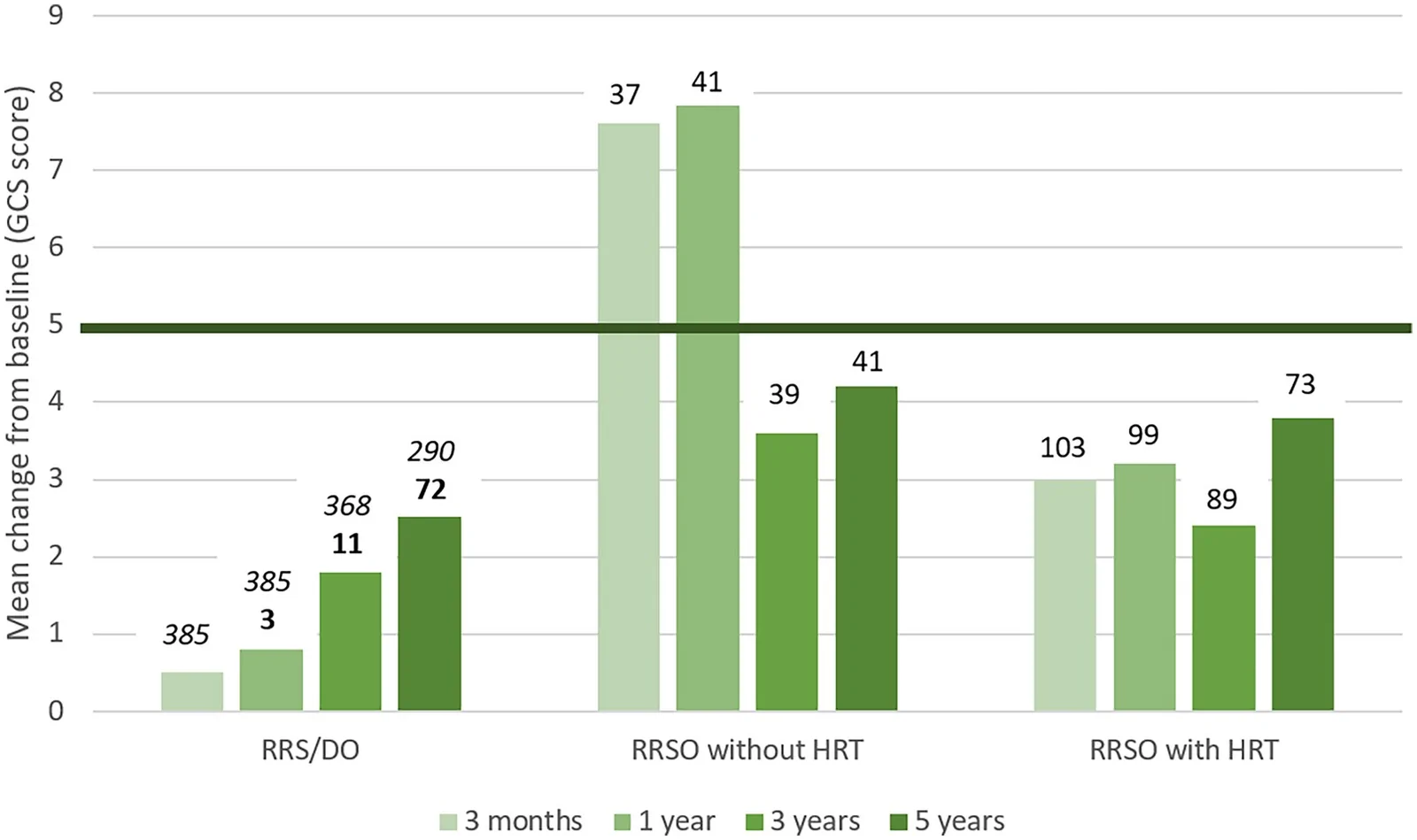
Mean change from baseline (GCS) across all follow-up time points. The **bold line** represents the average increase associated with natural, non-premature menopause in the Dutch population. Numbers in **italics** represent women who had RRS only; **bold numbers** represent the number of women that had both RRS and DO. Abbreviations: DO = delayed oophorectomy; HRT = hormone replacement therapy; RRS = risk-reducing salpingectomy; RRSO = risk-reducing salpingo-oophorectomy.

The primary outcome of the TUBA study was menopause-related QoL. We observed a mean (SD) increase from baseline GCS score of 2.6 (7.6) points in the RRS/DO group, compared with 4.2 (7.9) points in the RRSO without HRT group 5 years post-surgery (Figure 3; Table 2). The aMD between the RRS/DO and RRSO without HRT group was 1.8 point (95% CI = −0.6 to 4.3) higher in the RRS/DO group at 5-year follow-up. The aMD increased up to 1 year after surgery, indicating more menopausal complaints, after which it gradually decreased and was no longer statistically significant at 5 years follow-up (Table S2). The vasomotor and sexual subdomains showed differences in favor of RRS/DO (Table S3). When comparing RRS/DO to RRSO with HRT, the aMD was 1.4 point (95% CI = −0.4 to 3.1). The course of the mean change from baseline of both RRSO groups was similar, yet differences were less pronounced in the RRSO with HRT group (Figure 3). In the linear mixed model, the estimate for the RRS group compared with the RRSO without HRT group was −3.1 (standard error = 2.1, 95% CI = −7.2 to 0.9) and −2.3 (standard error = 1.5, 95% CI = −5.2 to 0.6) (Tables S4 and S5).

**Table 2.** Total score, change from baseline, and aMDs in the three separate groups (GCS) including sensitivity analysis, which excluded women who had both RRS and DO at the time of the follow-up moment.

	RRS/DO		RRSO without HRT				RRSO with HRT			
	N	Score	N	Score	aMD	95% CI	N	Score	aMD	95% CI
	Primary analysis									
Baseline, median (IQR)	404	6.0 (3.0; 11.8)	40	11.0 (5.3; 14.0)			107	7.0 (4.0; 12.0)		
At 5 years, median (IQR)	363	8.0 (4.0; 15.0)	41	15.0 (9.0; 18.0)			74	12.0 (7.0; 17.3)		
CFB at 5 years, *mean (SD)*	362	*2.6 (7.6)*	41	*4.2 (7.9)*	1.8	−0.6; 4.3	73	*3.8 (8.6)*	1.4	−0.4; 3.1
	Sensitivity analysis excluding women with both salpingectomy and oophorectomy									
Baseline, median (IQR)	404	6.0 (3.0; 11.8)	40	11.0 (5.3; 14.0)			107	7.0 (4.0; 12.0)		
At 5 years, median (IQR)	291	8.0 (3.0; 14.0)	41	15.0 (9.0; 18.0)			74	12.0 (7.0; 17.3)		
CFB at 5 years, *mean (SD)*	290	*1.7 (7.1)*	41	*4.2 (7.9)*	2.4	0.1; 4.8	73	*3.8 (8.6)*	1.9	0.1; 3.7

A higher score on The GCS (0-63) indicates more climacteric symptoms. Adjusting factors in the analysis: type of strategy (RRS/DO vs RRSO), type of *BRCA*-PV (*BRCA1* vs *BRCA2*), follow-up time point (3 months, 1, 3, and 5 years post-surgery), age at first surgery, baseline GCS score, baseline SF-36 physical component scores, a random effect for center of inclusion, an unstructured correlation matrix for the within-participants correlation between repeated measurements (3 months, 1, 3, and 5 years post-surgery), a three-way interaction for type of strategy by follow-up time point by baseline GCS score, and its corresponding 2-way interactions. Participants were classified into the RRSO with or without HRT group based on their HRT use at the time of the follow-up questionnaire.

Abbreviations: aMD = adjusted mean difference; CFB = change from baseline; CI = confidence interval; DO = delayed oophorectomy; HRT = hormone replacement therapy; IQR = interquartile range; N = number of participants; RRS = risk-reducing salpingectomy; RRSO = risk-reducing salpingo-oophorectomy; SD = standard deviation.Numbers in *italics* indicate is concerns a *mean (standard deviation)*

Overall, proportions of GCS scores that decreased (better outcomes), remained stable, or increased (worse outcomes) were comparable between all groups at 5-year follow-up (Figure S1).

In the first sensitivity analysis, we excluded women who underwent oophorectomy in the RRS/DO group. We observed a difference in change of menopause-related QoL 5 years post-surgery between the salpingectomy and both the RRSO without HRT (aMD = 2.4, 95% CI = 0.1 to 4.1) and RRSO with HRT (aMD = 1.9, 95% CI = 0.1 to 3.7) groups (Table 2). In the subdomains, differences were exclusively present in the vasomotor and sexual subdomains (Table S6). In the second sensitivity analysis, which excluded participants with a first surgery before the recommended guideline age, no notable differences with the primary analyses were observed.

### Secondary outcomes

We observed a significant deterioration in sexual functioning and sexual distress at all time points (compared with baseline) in the RRSO without HRT group compared with the whole RRS/DO group (Table S7). Differences were most pronounced 1-year post-surgery (FSFI with an aMD = −6.2, 95% CI = −8.4 to −4.1) and gradually reduced afterward. The salpingo-oophorectomy groups showed a greater increase in the proportion of participants with sexual dysfunction and sexual distress compared to the RRS/DO group during the complete follow-up (Figures S2 and S3).

Moreover, the salpingo-oophorectomy groups experienced higher reductions in cancer worry compared to the RRS/DO group (Table S7). In the RRS/DO group, cancer worry decreased after salpingectomy and stabilized afterward (Figure S4). Decisional regret slightly increased in the RRSO without HRT group (score from 5 to 10) and RRSO with HRT group (from 10 to 12.5) compared with the RRS/DO group (5 at baseline and 5-year follow-up) but remained in the mild/moderate category (DRS score = 5-25) (Table S7). For the health-related QoL, no differences in change from baseline score at 5 years post-surgery were observed regarding the physical component score. However, the mental component score showed a greater increase after RRSO without HRT compared with RRS/DO at 5-year follow-up, indicating a better mental health (aMD = 3.1, 95% CI = 0.4 to 5.7) (Table S7).

Restricting the secondary analyses to participants who underwent only salpingectomy and not oophorectomy did not change the results of secondary outcomes, except that the difference in the mental component of health-related QoL at 5 years was no longer observed (Table S8).

Surgical and histopathological outcomes were consistent with those reported in our previous study.^17^

## Discussion

### Main findings

The 5-year follow-up of the TUBA study evaluated menopause-related QoL in *BRCA1/2*-PV carriers after RRS/DO and compared it with RRSO with and without HRT. Our results suggest delaying oophorectomy plays an important role in mitigating the decrease in menopause-related QoL. We observed a difference in menopause-related QoL when comparing women after salpingectomy before oophorectomy to salpingo-oophorectomy (with and without HRT), indicating less decline in menopause-related QoL after salpingectomy. However, we did not observe this difference when comparing the strategy RRS/DO (with and without oophorectomy) with salpingo-oophorectomy (with and without HRT) 5 years after first surgery. Therefore, the oophorectomy appears to be the key factor in declining menopause-related QoL. Differences in QoL between RRS/DO and RRSO without HRT were more pronounced compared to RRS/DO and RRSO with HRT across the entire follow-up period.

We observed that there was no difference in menopause-related QoL between the RRS/DO and RRSO groups at 5-year follow-up, contrary to the 1- and 3-year results of this study.^16^^,^^17^ The lack of difference we observed can be explained by the 17.6% of women in the RRS/DO group who had their oophorectomy within 5 years after salpingectomy. When we compared women who underwent RRS without oophorectomy (yet) with salpingo-oophorectomy, the decrease in QoL was less in women after salpingectomy, indicating fewer climacteric symptoms. This implies that the oophorectomy has a higher adverse impact on QoL than the salpingectomy, and all pros and cons of oophorectomy should be considered when deciding about the timing of the intervention. This comparison is highly relevant for clinical practice, as it reflects real-world scenarios in which women had their RRS but not DO yet. Therefore, it is important for counseling, timing of interventions, and decision-making. The observed adverse effects on QoL after removal of the ovaries correspond with the findings of other studies.^24^^,^^25^ The limited influence of salpingectomy on QoL is also confirmed by Skorupska et al., who showed that a prophylactic salpingectomy at hysterectomy did not contribute to changes in QoL or sexual functioning.^26^ Therefore, postponing the oophorectomy appears to provide benefits in preventing deterioration of menopause-related QoL.

Women who underwent salpingectomy without oophorectomy (yet) had a mean increase in GCS score of 1.7 (SD = 7.1), reflecting more climacteric symptoms. This increase is limited compared with natural menopause, which corresponds to an increase of 5 points on the GCS scale in the general Dutch population.^18^ The small observed increase could be due to by increasing age. Interestingly, the mean (SD) increase in the RRSO without HRT group (4.2 [7.9]) and with HRT group (3.8 [8.6]) were also below 5 points, indicating they experience fewer climacteric symptoms compared with natural menopause.

We observed a decrease in HRT use and a change in the used HRT types during the follow-up period. Tibolone was the predominant type in the beginning, but its use gradually declined and was replaced by (transdermal) estradiol in combination with progesterone (oral or IUD). This reflects the recent changes in the national Dutch guideline on HRT medication, as this new combination is considered more effective with fewer side-effects due to its bio-identical nature.^27^^,^^28^ The type and dosage of estradiol and progesterone varied, reflecting the lack of knowledge on optimal HRT use. Initial uptake of HRT after DO (in women without prior breast cancer) was lower compared with after RRSO (65.4% vs 85.6%). This is interesting, as virtually all participants having DO were before age 50. Although exact considerations remain unknown, a possible explanation is that these women are closer to the age of natural menopause compared with women who chose RRSO and, therefore, feel less inclined to use HRT. This shows that there is a need for future research into this topic. Nonetheless, we showed that the use of HRT consistently mitigated the negative impact of salpingo-oophorectomy on QoL but did not eliminate it entirely. This is consistent with findings of other studies.^10^^,^^25^^,^^29^^,^^30^

To our knowledge, this is the first prospective study providing insights into the long-term QoL follow-up after RRS/DO and into the timing of oophorectomy after previous salpingectomy. The timing of oophorectomy varied substantially, ranging from within the recommended salpingo-oophorectomy guideline age to the upper limits permitted by the study. The vast majority of women (87.2%), who had their oophorectomy within 5 years after salpingectomy, had this within the guideline age range for salpingo-oophorectomy. Explanations for this could be that they still worried about cancer after salpingectomy, or that they planned to have salpingectomy before the guideline age and their oophorectomy within the guideline age, seeking more safety than current guidelines advice. The alternative strategy might still have delayed premature menopause in individual participants, as they could have postponed their oophorectomy beyond the moment they originally intended to undergo salpingo-oophorectomy. Important to note is that the executed delayed oophorectomies in this study are only a subsample and do not represent the complete study population or the full follow-up time. Women who have not yet undergone their DO may have chosen to wait until the maximum recommended age, which they have not yet reached. Therefore, we expect the interval between salpingectomy and oophorectomy of the whole group to be larger than reported in this article.

The main strengths include the prospective design, large number of participants, and nationwide coverage. Moreover, standardized and validated questionnaires and cutoff values were used. Response rates were high, which increased validity and generalizability. Furthermore, we used a preferential design. Although it is not a standard randomized controlled trial, previous studies have shown that a randomized controlled trial would have been unfeasible because of the diverse consequences of each strategy.^31^^,^^32^ The chosen preferential design enables investigation of challenging research questions. Last, the safety rule and regular meetings with the data safety monitoring board enabled real-time safety monitoring of the study.

Limitations consist of possible selection bias due to RRS/DO only being available in participating centers, whereas RRSO can also be performed in (smaller) nonparticipating hospitals. Furthermore, HRT use was self-reported. Participants may have initiated or discontinued HRT shortly before completing the questionnaire, potentially leading to misclassification into a group that did not accurately reflect their hormonal status at that time.

Important to note is that the oncological safety of this alternative approach has not been established yet. TUBA-WISP II (NCT04294927),^16^ SoROCK (NCT04251052),^33^ and PROTECTOR (ISRCTN25173360)^34^ aim to evaluate this, as proof of oncological safety is a prerequisite to integrate this approach into clinical practice.

## Conclusion

Our study showed that delaying oophorectomy appears to be a key factor in preventing deterioration in menopause-related QoL. The decline in menopause-related QoL was smaller in women after salpingectomy (without oophorectomy), compared with salpingo-oophorectomy, whereas it was similar when comparing all women choosing salpingectomy with DO compared with salpingo-oophorectomy (with and without HRT) 5 years after the first surgery.

## Supplementary Material

djag110_Supplementary_Data

## Data Availability

The data analyzed in this study are subject to privacy and ethical considerations and are not immediately available for public access due to laws from the *Dutch Data Protection Authority* (Autoriteit Persoonsgegevens). De-identified patient data used for this article will be made available on request for academic use, within the limitations of the informed consent and the study’s consortium agreement. The data can be accessed through a formal application process. Interested parties may apply for access to the data by submitting a formal application that includes their contact information and the purpose for which the data is required. Requests can be sent to tuba-wisp@radboudumc.nl and will be reviewed in accordance with ethical and legal considerations. Data access will be granted if the application complies with the privacy regulations outlined by the Dutch Data Protection Authority. All requests will be handled by the steering committee of the TUBA study, and access will be granted based on the criteria established *Dutch Data Protection Authority.* A response will be provided within 60 days.
