# Supplementary material for: Menopause-related quality of life 5 years after salpingectomy with delayed oophorectomy vs salpingo-oophorectomy
Source: J Natl Cancer Inst. 2026 Apr 7;118(9):1731–40. doi: 10.1093/jnci/djag110 (PMC13549723; doi:10.1093/jnci/djag110)
Supplement: Supplementary file 1 [file djag110_supplementary_data.docx]

**Supplementary material**

| **Table S1 Overview of hormone replacement therapy after oophorectomy** | | | | | |
| --- | --- | --- | --- | --- | --- |
|  | | Three months | One year | Three years | Five years |
|  | | N = 104 | N = 101 | N = 98 | N = 109 |
| **Estrogen/progestogen** | |  |  |  |  |
| **Tibolone** | |  | | | |
|  | 2.5 mg | 60 | 54 | 50 | 40 |
| **Estradiol/dydrogesteron** | |  | | | |
|  | 1 mg / 5 mg | 14 | 15 | 13 | 20 |
|  | 1 mg / 10 mg | 3 |  | 1 | 1 |
|  | 2 mg / 10 mg | 1 | 4 | 2 | 1 |
|  | Dose unknown | 7 | 9 | 8 | 11 |
| **Estradiol/drospirenone** | |  |  |  |  |
|  | 1 mg / 2 mg | 1 | 1 |  |  |
| **Estradiol/norethisterone** | |  |  |  |  |
|  | Dose unknown / dose unknown |  |  | 1 |  |
| **Estradiol/** **LNG-IUD 52 mg** | |  |  |  |  |
|  | 1 mg | 1 | 1 | 1 |  |
| **Estradiol/unknown** | |  |  |  |  |
|  | Dose unknown |  |  | 1 | 1 |
| **Estradiol patch/progesterone** | |  | | | |
|  | 37.5 µg / 200 mg | 1 |  |  |  |
|  | 50 µg / 100 mg | 2 | 2 | 3 | 2 |
|  | 50 µg / 200 mg | 1 | 2 | 2 | 1 |
|  | 50 µg / dose unknown |  | 1 |  |  |
|  | 75 µg / 100 mg |  | 1 |  |  |
|  | 75 µg / 200 mg |  |  |  | 3 |
|  | Dose unknown / 100 mg |  |  |  | 1 |
|  | Dose unknown / 200 mg | 1 | 1 |  |  |
|  | Dose unknown / dose unknown | 2 | 1 |  | 1 |
| **Estradiol patch/** **LNG-IUD 52 mg** | |  |  |  |  |
|  | 50 µg |  |  |  | 1 |
|  | 75 µg |  |  | 1 | 1 |
|  | Dose unknown |  |  | 1 |  |
| **Estradiol patch/dydrogesteron** | |  |  |  |  |
|  | Dose unknown / 10 mg |  |  |  | 1 |
| **Estradiol patch/unknown** | |  |  |  |  |
|  | 50 µg |  |  | 1 | 2 |
|  | 75 µg |  |  |  | 1 |
|  | Dose unknown | 1 | 1 | 1 |  |
| **Estradiol spray/progesterone** | |  |  |  |  |
|  | 1.53 mg / 100 mg |  |  | 1 |  |
|  | 1.53 mg / 200 mg |  |  | 1 | 1 |
|  | 1.53 mg / dose unknown |  |  | 1 | 1 |
|  | 3.06 mg / 100 mg |  | 1 | 1 | 1 |
|  | 4.59 mg / 200 mg |  |  | 1 | 2 |
|  | Dose unknown / dose unknown |  |  | 1 | 1 |
| **Estradiol spray/unknown** | |  |  |  |  |
|  | 1.53 mg |  |  | 1 |  |
|  | 4.59 mg |  |  |  | 1 |
| **Estradiol gel/progesterone** | |  |  |  |  |
|  | 0.75 mg / 100 mg |  |  |  | 3 |
| **Ethinylestradiol/desogestrel ^a^** | |  |  |  |  |
|  | 0.02 mg / 0.15 mg | 1 | 1 | 1 |  |
| **Off label medication** | |  |  |  | 1 |
| **Unknown/progesterone** | |  |  |  |  |
|  | 100 mg |  |  | 1 |  |
|  | Dose unknown |  |  | 1 | 2 |
| **Unknown/ LNG-IUD 52 mg** | |  |  | 1 |  |
| **Unknown/unknown** | | 8 | 6 | 1 | 8 |

Abbreviations: N number of participants; mg milligram; µg microgram.

^a^ Oral contraceptive after removal of the ovaries.

| **Table S2. Total score, change from baseline and adjusted mean differences (Greene Climacteric Scale) in the three different groups including sensitivity analysis which excluded women who had both RRS and DO at the time of the follow-up moment** | | | | | | | | | | | |
| --- | --- | --- | --- | --- | --- | --- | --- | --- | --- | --- | --- |
| **Primary analysis** | | | | | | | | | | | |
|  | **RRS/DO** | | | **RRSO without HRT** | | | | **RRSO with HRT** | | | |
|  | N | Median (IQR)  Mean (SD) | | N | Median (IQR)  Mean (SD) | aMD | 95% CI | N | Median (IQR)  Mean (SD) | aMD | 95% CI |
| Baseline score | 404 | 6.0 (3.0; 11.8) | | 40 | 11.0 (5.3; 14.0) |  |  | 107 | 7.0 (4.0; 12.0) |  |  |
| Score at 3 months | 385 | 6.0 (2.0; 12.0) | | 37 | 19.0 (13.5; 24.0) |  |  | 104 | 11.0 (7.0; 16.0) |  |  |
| CFB at 3 months | 382 | 0.5 (5.5) | | 37 | 7.6 (6.8) | 7.6 | **5.6; 9.6** | 103 | 3.5 (6.7) | 3.0 | **1.8; 4.3** |
| Score at 1 year | 389 | 7.0 (3.0; 12.0) | | 42 | 17.5 (12.8; 24.5) |  |  | 100 | 12.0 (6.0; 18.0) |  |  |
| CFB at 1 year | 388 | 0.8 (6.1) | | 41 | 7.8 (8.7) | 8.4 | **6.4; 10.4** | 99 | 4.1 (7.0) | 3.2 | **1.9; 4.6** |
| Score at 3 years | 381 | 8.0 (4.0; 13.0) | | 40 | 14.0 (10.0; 19.0) |  |  | 90 | 12.0 (7.0; 19.0) |  |  |
| CFB at 3 years | 379 | 1.8 (6.5) | | 39 | 3.6 (8.5) | 3.8 | **1.7; 6.0** | 89 | 4.4 (6.8) | 2.4 | **0.9; 3.9** |
| **Sensitivity analysis excluding women with both RRS and DO** | | | | | | | | | | | |
|  | **RRS without DO** | | | **RRSO without HRT** | | | | **RRSO with HRT** | | | |
|  | N | Median (IQR)  Mean (SD) | *p*-value | N | Median (IQR)  Mean (SD) | aMD | 95% CI | N | Median (IQR)  Mean (SD) | aMD | 95% CI |
| Baseline score | 404 | 6.0 (3.0; 11.8) | | 40 | 11.0 (5.3; 14.0) |  |  | 107 | 7.0 (4.0; 12.0) |  |  |
| Score at 3 months | 385 | 6.0 (2.0; 12.0) | | 37 | 19.0 (13.5; 24.0) |  |  | 104 | 11.0 (7.0; 16.0) |  |  |
| CFB at 3 months | 382 | 0.5 (5.5) | | 37 | 7.6 (6.8) | 7.7 | **5.6; 9.8** | 103 | 3.5 (6.7) | 2.7 | **1.4; 4.0** |
| Score at 1 year | 386 | 7.0 (3.0; 12.0) | | 42 | 17.5 (12.8; 24.5) |  |  | 100 | 12.0 (6.0; 18.0) |  |  |
| CFB at 1 year | 385 | 0.7 (6.1) | | 41 | 7.8 (8.7) | 7.7 | **5.6; 9.8** | 99 | 4.1 (7.0) | 3.1 | **1.7; 4.4** |
| Score at 3 years | 370 | 8.0 (4.0; 13.0) | | 40 | 14.0 (10.0; 19.0) |  |  | 90 | 12.0 (7.0; 19.0) |  |  |
| CFB at 3 years | 368 | 1.6 (6.5) | | 39 | 3.6 (8.5) | 4.3 | **2.1; 6.5** | 89 | 4.4 (6.8) | 2.0 | **0.5; 3.5** |

A higher score on The Greene Climacteric Scale (0-63) indicates more climacteric symptoms. Adjusting factors in the analysis: type of strategy (RRS/DO versus RRSO), type of BRCA-PV (*BRCA1* versus *BRCA2*), follow-up time point (3 months, 1, 3 and 5 years post-surgery), age at first surgery, baseline GCS score, baseline SF-36 physical component scores, a random effect for centre of inclusion, an unstructured correlation matrix for the within-participants correlation between repeated measurements (3 months, 1, 3 and 5 years post-surgery), a three-way interaction for type of strategy by follow-up time point by baseline GCS score, and its corresponding two-way interactions. Participants were classified into the RRSO with or without HRT group based on their HRT use at the time of the follow-up questionnaire.

Abbreviations: RRS risk-reducing salpingectomy; DO delayed oophorectomy; RRSO risk-reducing salpingo-oophorectomy; HRT hormone replacement therapy; N number of participants; IQR Interquartile Range; SD standard deviation; aMD adjusted mean difference; CI confidence interval; CFB change from baseline.

| **Table S3. Scores for the subdomains of the Greene Climacteric Scale** | | | | | |  |  |  |  |  |  |
| --- | --- | --- | --- | --- | --- | --- | --- | --- | --- | --- | --- |
|  | | **RRS/DO** | | **RRSO without HRT** | | | | **RRSO with HRT** | | | |
|  |  | N | Median (IQR)  Mean (SD) | N | Median (IQR)  Mean (SD) | aMD | 95% CI | N | Median (IQR)  Mean (SD) | aMD | 95% CI |
| **Anxiety ^a^**  *Range 0-18* | Baseline score | 404 | 2.0 (1.0; 4.0) | 40 | 3.0 (1.3; 5.0) |  |  | 107 | 2.0 (1.0; 4.0) |  |  |
|  | Score at 3 months | 385 | 2.0 (0.0; 4.0) | 37 | 6.0 (4.0; 8.0) |  |  | 104 | 3.0 (2.0; 5.0) |  |  |
|  | CFB at 3 months | 382 | 0.1 (2.2) | 37 | 2.4 (2.8) | 2.2 | **1.5; 3.0** | 103 | 0.7 (2.4) | 0.6 | **0.2; 1.1** |
|  | Score at 1 year | 389 | 2.0 (0.0; 4.0) | 42 | 5.0 (3.0; 8.0) |  |  | 100 | 3.5 (1.0; 5.0) |  |  |
|  | CFB at 1 year | 388 | 0.2 (2.3) | 41 | 1.8 (3.2) | 1.9 | **1.2; 2.6** | 99 | 1.1 (2.8) | 0.9 | **0.4; 1.4** |
|  | Score at 3 years | 382 | 2.0 (1.0; 4.0) | 40 | 4.0 (2.0; 6.8) |  |  | 90 | 3.0 (2.0; 5.3) |  |  |
|  | CFB at 3 years | 380 | 0.5 (2.4) | 39 | 0.7 (3.3) | 1.0 | **0.2; 1.7** | 89 | 1.2 (2.8) | 0.7 | **0.1; 1.2** |
|  | Score at 5 years | 363 | 2.0 (1.0; 5.0) | 41 | 4.0 (2.0; 6.0) |  |  | 74 | 3.0 (2.0; 5.0) |  |  |
|  | CFB at 5 years | 362 | 0.7 (2.7) | 41 | 1.0 (3.1) | 0.5 | -0.3; 1.3 | 73 | 1.0 (3.0) | 0.3 | -0.4; 0.9 |
| **Depression ^b^**  *Range 0-15* | Baseline score | 404 | 2.0 (1.0; 4.0) | 40 | 3.0 (2.0; 5.0) |  |  | 107 | 2.0 (1.0; 4.0) |  |  |
|  | Score at 3 months | 385 | 2.0 (1.0; 4.0) | 37 | 5.0 (3.0; 7.0) |  |  | 104 | 3.0 (2.0; 5.0) |  |  |
|  | CFB at 3 months | 382 | 0.1 (2.1) | 37 | 1.1 (2.3) | 1.3 | **0.6; 2.1** | 103 | 0.7 (2.4) | 0.4 | 0.0; 0.9 |
|  | Score at 1 year | 389 | 2.0 (1.0; 4.0) | 42 | 5.0 (3.0; 7.0) |  |  | 100 | 3.0 (1.0; 5.0) |  |  |
|  | CFB at 1 year | 388 | 0.1 (2.3) | 41 | 1.1 (2.8) | 1.5 | **0.8; 2.2** | 99 | 0.8 (3.0) | 0.5 | 0.0; 1.0 |
|  | Score at 3 years | 382 | 2.0 (1.0; 4.0) | 40 | 3.0 (1.0; 5.8) |  |  | 90 | 2.5 (1.0; 5.0) |  |  |
|  | CFB at 3 years | 380 | 0.3 (2.4) | 39 | -0.3 (2.6) | -0.1 | -0.9; 0.6 | 89 | 0.7 (2.7) | 0.3 | -0.2; 0.8 |
|  | Score at 5 years | 363 | 2.0 (1.0; 5.0) | 41 | 3.0 (1.0; 5.0) |  |  | 74 | 3.0 (1.0; 5.0) |  |  |
|  | CFB at 5 years | 362 | 0.4 (2.7) | 41 | -0.3 (2.8) | -0.4 | -1.2; 0.4 | 73 | 0.8 (3.2) | 0.3 | -0.3; 0.9 |
| **Somatic ^c^**  *Range 0-21* | Baseline score | 404 | 1.0 (0.0; 3.0) | 40 | 2.0 (1.0; 3.0) |  |  | 107 | 1.0 (1.0; 3.0) |  |  |
|  | Score at 3 months | 385 | 2.0 (0.0; 3.0) | 37 | 4.0 (2.0; 7.0) |  |  | 104 | 2.0 (1.0; 4.0) |  |  |
|  | CFB at 3 months | 382 | 0.3 (2.1) | 37 | 1.7 (2.4) | 1.4 | **0.8; 2.1** | 103 | 1.0 (2.5) | 0.8 | **0.4; 1.3** |
|  | Score at 1 year | 389 | 2.0 (0.0; 3.0) | 42 | 4.5 (3.0; 6.0) |  |  | 100 | 2.5 (1.0; 4.8) |  |  |
|  | CFB at 1 year | 388 | 0.3 (2.2) | 41 | 2.0 (3.0) | 1.6 | **0.9; 2.3** | 99 | 1.1 (2.0) | 0.7 | **0.3; 1.2** |
|  | Score at 3 years | 382 | 2.0 (1.0; 4.0) | 40 | 4.0 (2.0; 5.0) |  |  | 90 | 3.0 (1.0; 5.0) |  |  |
|  | CFB at 3 years | 380 | 0.6 (2.4) | 39 | 1.2 (2.7) | 0.7 | -0.1; 1.4 | 89 | 1.1 (2.4) | 0.5 | 0.0; 1.0 |
|  | Score at 5 years | 363 | 2.0 (1.0; 4.0) | 41 | 3.0 (1.0; 5.0) |  |  | 74 | 3.0 (1.0; 5.0) |  |  |
|  | CFB at 5 years | 362 | 0.7 (2.5) | 41 | 1.1 (3.2) | 0.4 | -0.4; 1.1 | 73 | 1.0 (2.5) | 0.3 | -0.2; 0.9 |
| **Vasomotor ^d^**  *Range 0-6* | Baseline score | 404 | 0.0 (0.0; 0.0) | 40 | 0.0 (0.0; 0.0) |  |  | 107 | 0.0 (0.0; 1.0) |  |  |
|  | Score at 3 months | 385 | 0.0 (0.0; 0.0) | 37 | 2.0 (2.0; 3.0) |  |  | 104 | 2.0 (0.3; 2.0) |  |  |
|  | CFB at 3 months | 382 | 0.0 (0.7) | 37 | 2.1 (1.2) | 2.0 | **1.8; 2.3** | 103 | 1.0 (1.1) | 1.1 | **0.9; 1.3** |
|  | Score at 1 year | 389 | 0.0 (0.0; 1.0) | 42 | 2.0 (2.0; 4.0) |  |  | 100 | 1.0 (0.0; 2.0) |  |  |
|  | CFB at 1 year | 388 | 0.1 (0.8) | 41 | 2.1 (1.4) | 2.0 | **1.7; 2.3** | 99 | 0.8 (1.1) | 0.8 | **0.6; 1.0** |
|  | Score at 3 years | 382 | 0.0 (0.0; 1.0) | 40 | 2.0 (1.0; 2.8) |  |  | 90 | 1.0 (0.0; 2.0) |  |  |
|  | CFB at 3 years | 380 | 0.3 (1.0) | 39 | 1.2 (1.6) | 1.2 | **0.8; 1.6** | 89 | 1.0 (1.2) | 0.7 | **0.5; 1.0** |
|  | Score at 5 years | 363 | 0.0 (0.0; 1.0) | 41 | 2.0 (1.0; 3.0) |  |  | 74 | 1.0 (0.0; 2.0) |  |  |
|  | CFB at 5 years | 362 | 0.5 (1.2) | 41 | 1.6 (1.4) | 0.8 | **0.4; 1.3** | 73 | 0.6 (1.4) | 0.3 | 0.0; 0.6 |
| **Sexual ^e^**  *Range 0-3* | Baseline score | 404 | 0.0 (0.0; 1.0) | 40 | 1.0 (0.0; 1.0) |  |  | 107 | 0.0 (0.0; 1.0) |  |  |
|  | Score at 3 months | 385 | 0.0 (0.0; 1.0) | 37 | 1.0 (0.0; 2.0) |  |  | 104 | 0.5 (0.0; 1.0) |  |  |
|  | CFB at 3 months | 382 | 0.0 (0.7) | 37 | 0.3 (0.9) | 0.4 | **0.1; 0.6** | 103 | 0.1 (0.9) | 0.1 | -0.1; 0.3 |
|  | Score at 1 year | 389 | 0.0 (0.0; 1.0) | 42 | 2.0 (1.0; 2.0) |  |  | 100 | 1.0 (0.0; 1.0) |  |  |
|  | CFB at 1 year | 388 | 0.0 (0.7) | 41 | 0.9 (0.9) | 0.8 | **0.5; 1.0** | 99 | 0.2 (0.9) | 0.3 | **0.1; 0.4** |
|  | Score at 3 years | 381 | 0.0 (0.0; 1.0) | 40 | 2.0 (1.0; 2.0) |  |  | 90 | 1.0 (0.0; 2.0) |  |  |
|  | CFB at 3 years | 379 | 0.2 (0.9) | 39 | 0.8 (0.9) | 0.6 | **0.3; 0.9** | 89 | 0.4 (1.0) | 0.3 | 0.0; 0.5 |
|  | Score at 5 years | 363 | 1.0 (0.0; 1.0) | 41 | 1.0 (1.0; 2.0) |  |  | 74 | 1.0 (0.0; 2.0) |  |  |
|  | CFB at 5 years | 362 | 0.3 (0.9) | 41 | 0.7 (1.0) | 0.5 | **0.1; 0.8** | 73 | 0.4 (1.0) | 0.4 | 0.0; 0.5 |

Participants were classified into the RRSO group with or without HRT at each time point, based on their HRT use at that time.

Abbreviations: RRS risk-reducing salpingectomy; DO delayed oophorectomy; RRSO risk-reducing salpingo-oophorectomy; HRT hormone replacement therapy; N number of participants; IQR interquartile range; SD standard deviation; aMD adjusted mean difference; CI confidence interval; CFB change from baseline; ref reference.

^a^ Adjusting factors in the analysis: type of strategy, type of *BRCA*-PV, follow-up time point, age at first surgery, baseline GCS score, baseline GCS anxiety domain score, baseline SF-36 physical component score, a random effect for centre of inclusion, an unstructured correlation matrix for the within-participants correlation between repeated measurements, a three-way interaction for type of strategy by follow-up time point by baseline GCS anxiety domain score, and its corresponding two-way interactions.

^b^ Adjusting factors in the analysis: type of strategy, type of *BRCA*-PV, follow-up time point, age at first surgery, baseline GCS score, baseline GCS depression domain score, baseline SF-36 physical component score, baseline SF-36 mental component score, a random effect for centre of inclusion, an unstructured correlation matrix for the within-participants correlation between repeated measurements, a three-way interaction for type of strategy by follow-up time point by baseline GCS depression domain score, and its corresponding two-way interactions.

^c^ Adjusting factors in the analysis: type of strategy, type of *BRCA*-PV, follow-up time point, age at first surgery, baseline GCS score, baseline GCS somatic domain score, baseline SF-36 physical component score, a random effect for centre of inclusion, an unstructured correlation matrix for the within-participants correlation between repeated measurements, a two-way interaction for type of strategy by follow-up time point.

^d^ Adjusting factors in the analysis: type of strategy, type of *BRCA*-PV, follow-up time point, age at first surgery, baseline GCS score, baseline GCS vasomotor domain score, a random effect for centre of inclusion, an unstructured correlation matrix for the within-participants correlation between repeated measurements, a three-way interaction for type of strategy by follow-up time point by baseline GCS vasomotor domain score, a three-way interaction for type of strategy by follow-up time point by age at first surgery, and its corresponding two-way interactions.

^e^ Adjusting factors in the analysis: type of strategy, type of *BRCA*-PV, follow-up time point, age at first surgery, baseline GCS score, baseline GCS sexual domain score, a random effect for centre of inclusion, an unstructured correlation matrix for the within-participants correlation between repeated measurements, a three-way interaction for type of strategy by follow-up time point by baseline GCS sexual domain score, a three-way interaction for type of strategy by follow-up time point by age at first surgery, and its corresponding two-way interactions.

**Figure S1. Proportions of participants that decreased, did not change, or increased in total Greene Climacteric Scale score per time period**

A better score indicates a decrease in total GCS score, a worse score indicates an increase in total GCS-score.

Abbreviations: RRS risk-reducing salpingectomy; DO delayed oophorectomy; RRSO risk-reducing salpingo-oophorectomy; HRT hormone replacement therapy.

**Table S4. Linear mixed model used for the difference in menopause-related quality of life between baseline and five-year follow-up in women after RRS/DO versus RRSO without HRT**

| **Predictor/interaction** | **Estimate (β)** | **Standard error** | **95% confidence interval** |
| --- | --- | --- | --- |
| **Intercept** | 11.5 | 3.9 | 3.9;19.2 |
| **Type of strategy**  RRS/DO  RRSO | -3.1  Ref | 2.1  NA | -7.2;0.9  NA |
| **Baseline value SF36 physical component score** | -0.1 | 0.0 | -0.2;-0.3 |
| **Visits**  1  2  3  4 | 2.4  6.3  2.2  Ref | 2.2  2.0  2.0  NA | -2.0;6.7  2.3;10.3  -1.7;6.2  NA |
| **Type of pathogenic variant**  *BRCA1*  *BRCA2* | -0.2  Ref | 2.1  NA | -1.1;0.7  NA |
| **Age at first surgery** | 0.1 | 0.1 | -0.1;0.2 |
| **Baseline value GCS score** | -0.5 | 0.2 | -0.8;-0.2 |
| **Visit * Type of strategy**  Visit 1 * RRS/DO  Visit 2 *RRS/DO  Visit 3 * RRS/DO  Visit 4 *RRS/DO  Visit 1 * RRSO  Visit 2 *RRSO  Visit 3 * RRSO  Visit 4 *RRSO | -5.5  -8.4  -3.0  Ref  Ref  Ref  Ref  Ref | 2.3  2.1  2.1  NA  NA  NA  NA  NA | -9.9;1.0  -12.6;-4.2  -7.2;1.0  NA  NA  NA  NA  NA |
| **Type of strategy * Baseline GCS score**  RRS/DO * Baseline value GCS score  RRSO * Baseline value GCS score | 0.2  Ref | 0.2  NA | -0.2;0.5  NA |
| **Visit * Type of Strategy * Baseline GCS score**  Visit 1 * RRS/DO *Baseline value GCS score  Visit 2 *RRS/DO * Baseline value GCS score  Visit 3 * RRS/DO * Baseline value GCS score  Visit 4 *RRS/DO * Baseline value GCS score  Visit 1 * RRSO * Baseline value GCS score  Visit 2 *RRSO * Baseline value GCS score  Visit 3 * RRSO * Baseline value GCS score  Visit 4 *RRSO * Baseline value GCS score | 0.1  0.0  0.0  Ref  0.2  -0.2  -0.1  Ref | 0.1  0.1  0.1  NA  0.2  0.2  0.1  NA | 0.0;0.2  -0.1;0.1  -0.1;0.1  NA  -0.2;0.5  -0.5;0.1  -0.4;0.2  NA |

Abbreviations: SF36 Short Form Health Survey 36; RRS risk-reducing salpingectomy; DO delayed oophorectomy; RRSO risk-reducing salpingectomy; GCS greene climacteric scale.

**Table S5. Linear mixed model used for the primary outcome in the comparison between RRS/DO and RRSO with HRT**

| **Predictor/interaction** | **Estimate (β)** | **Standard error** | **95% confidence interval** |
| --- | --- | --- | --- |
| **Intercept** | **9.8** | **3.5** | **2.8;16.7** |
| **Type of strategy**  RRS/DO  RRSO | -2.3  Ref | 1.5  NA | -5.2;0.6  NA |
| **Baseline value SF36 physical component score** | -0.1 | 0.0 | -0.1;0.0 |
| **Visits**  1  2  3  4 | 0.8  0.1  0.4  Ref | 1.4  1.3  1.3  NA | -3.4;1.9  -2.5;2.6  -2.9;2.1 |
| **Type of pathogenic variant**  *BRCA1*  *BRCA2* | -0.1  Ref | 0.5 | -1.0;0.8 |
| **Age at first surgery** | 0.0 | 0.1 | -0.1;0.2 |
| **Baseline value GCS score** | **-0.5** | **0.1** | **-0.7;-0.2** |
| **Visit * Type of strategy**  Visit 1 * RRS/DO  Visit 2 *RRS/DO  Visit 3 * RRS/DO  Visit 4 *RRS/DO  Visit 1 * RRSO  Visit 2 *RRSO  Visit 3 * RRSO  Visit 4 *RRSO | -2.3  -2.1  -0.4  Ref  Ref  Ref  Ref  Ref | 1.5  1.4  1.4  NA  NA  NA  NA  NA | -5.2;0.6  -4.9;0.6  -3.1;2.3  NA  NA  NA  NA  NA |
| **Type of strategy * Baseline GCS score**  RRS/DO * Baseline value GCS score  RRSO * Baseline value GCS score | **0.1**  Ref | **0.1**  NA | **0.0;0.2**  NA |
| **Visit * Type of Strategy * Baseline GCS score**  Visit 1 * RRS/DO *Baseline value GCS score  Visit 2 *RRS/DO * Baseline value GCS score  Visit 3 * RRS/DO * Baseline value GCS score  Visit 4 *RRS/DO * Baseline value GCS score  Visit 1 * RRSO * Baseline value GCS score  Visit 2 *RRSO * Baseline value GCS score  Visit 3 * RRSO * Baseline value GCS score  Visit 4 *RRSO * Baseline value GCS score | **0.1**  0.0  0.0  Ref  0.1  0.0  0.1  Ref | **0.1**  0.1  0.1  NA  0.1  0.1  0.1  NA | **0.0;0.2**  -0.1;0.1  -0.1;0.1  NA  -0.2;0.3  -0.2;0.2  -0.1;0.3  NA |

Abbreviations: SF36 Short Form Health Survey 36; RRS risk-reducing salpingectomy; DO delayed oophorectomy; RRSO risk-reducing salpingectomy; GCS greene climacteric scale.

| **Table S6. Sensitivity analyses of the subdomains of the Greene Climacteric Scale, excluding women after both RRS and DO** | | | | | | | | | | | | |
| --- | --- | --- | --- | --- | --- | --- | --- | --- | --- | --- | --- | --- |
|  | | **RRS without DO** | | | **RRSO without HRT** | | | | **RRSO with HRT** | | | |
|  |  | N | Median (IQR)  Mean (SD) |  | N | Median (IQR)  Mean (SD) | aMD | 95% Cl | N | Median (IQR)  Mean (SD) | aMD | 95% CI |
| **Anxiety**  *Range 0-18* | Baseline score | 404 | 2.0 (1.0; 4.0) | | 40 | 3.0 (1.3; 5.0) |  |  | 107 | 2.0 (1.0; 4.0) |  |  |
|  | Score at 3 months | 385 | 2.0 (0.0; 4.0) | | 37 | 6.0 (4.0; 8.0) |  |  | 104 | 3.0 (2.0; 5.0) |  |  |
|  | CFB at 3 months | 382 | 0.1 (2.2) | | 37 | 2.4 (2.8) | 2.2 | **1.5; 3.0** | 103 | 0.7 (2.4) | 0.6 | **0.2; 1.1** |
|  | Score at 1 year | 386 | 2.0 (0.0; 4.0) | | 42 | 5.0 (3.0; 8.0) |  |  | 100 | 3.5 (1.0; 5.0) |  |  |
|  | CFB at 1 year | 385 | 0.2 (2.3) | | 41 | 1.8 (3.2) | 1.9 | **1.2; 2.6** | 99 | 1.1 (2.8) | 0.9 | **0.4; 1.4** |
|  | Score at 3 years | 370 | 2.0 (1.0; 4.0) | | 40 | 4.0 (2.0; 6.8) |  |  | 90 | 3.0 (2.0; 5.3) |  |  |
|  | CFB at 3 years | 368 | 0.4 (2.4) | | 39 | 0.7 (3.3) | 1.0 | **0.2; 1.8** | 89 | 1.2 (2.8) | 0.7 | **0.2; 1.2** |
|  | Score at 5 years | 291 | 2.0 (1.0; 4.0) | | 41 | 4.0 (2.0; 6.0) |  |  | 74 | 3.0 (2.0; 5.0) |  |  |
|  | CFB at 5 years | 290 | 0.4 (2.5) | | 41 | 1.0 (3.1) | 0.7 | -0.1; 1.5 | 73 | 1.0 (3.0) | 0.5 | -0.1; 1.1 |
| **Depression**  *Range 0-15* | Baseline score | 404 | 2.0 (1.0; 4.0) | | 40 | 3.0 (2.0; 5.0) |  |  | 107 | 2.0 (1.0; 4.0) |  |  |
|  | Score at 3 months | 385 | 2.0 (1.0; 4.0) | | 37 | 5.0 (3.0; 7.0) |  |  | 104 | 3.0 (2.0; 5.0) |  |  |
|  | CFB at 3 months | 382 | 0.1 (2.1) | | 37 | 1.1 (2.3) | 1.3 | **0.6; 2.1** | 103 | 0.7 (2.4) | 0.4 | 0.0; 0.9 |
|  | Score at 1 year | 386 | 2.0 (1.0; 4.0) | | 42 | 5.0 (3.0; 7.0) |  |  | 100 | 3.0 (1.0; 5.0) |  |  |
|  | CFB at 1 year | 385 | 0.1 (2.3) | | 41 | 1.1 (2.8) | 1.5 | **0.8; 2.2** | 99 | 0.8 (3.0) | 0.5 | 0.0; 1.0 |
|  | Score at 3 years | 370 | 2.0 (1.0; 4.0) | | 40 | 3.0 (1.0; 5.8) |  |  | 90 | 2.5 (1.0; 5.0) |  |  |
|  | CFB at 3 years | 368 | 0.3 (2.4) | | 39 | -0.3 (2.6) | -0.1 | -0.9; 0.6 | 89 | 0.7 (2.7) | 0.3 | -0.2; 0.9 |
|  | Score at 5 years | 291 | 2.0 (1.0; 4.0) | | 41 | 3.0 (1.0; 5.0) |  |  | 74 | 3.0 (1.0; 5.0) |  |  |
|  | CFB at 5 years | 290 | 0.2 (2.6) | | 41 | -0.3 (2.8) | -0.3 | -1.1; 0.5 | 73 | 0.8 (3.2) | 0.4 | -0.2; 1.0 |
| **Somatic**  *Range 0-21* | Baseline score | 404 | 1.0 (0.0; 3.0) | | 40 | 2.0 (1.0; 3.0) |  |  | 107 | 1.0 (1.0; 3.0) |  |  |
|  | Score at 3 months | 385 | 2.0 (0.0; 3.0) | | 37 | 4.0 (2.0; 7.0) |  |  | 104 | 2.0 (1.0; 4.0) |  |  |
|  | CFB at 3 months | 382 | 0.3 (2.1) | | 37 | 1.7 (2.4) | 1.4 | **0.8; 2.1** | 103 | 1.0 (2.5) | 0.8 | **0.4; 1.3** |
|  | Score at 1 year | 386 | 2.0 (0.0; 3.0) | | 42 | 4.5 (3.0; 6.0) |  |  | 100 | 2.5 (1.0; 4.8) |  |  |
|  | CFB at 1 year | 385 | 0.3 (2.2) | | 41 | 2.0 (3.0) | 1.6 | **1.0; 2.3** | 99 | 1.1 (2.0) | 0.8 | **0.3; 1.2** |
|  | Score at 3 years | 370 | 2.0 (1.0; 4.0) | | 40 | 4.0 (2.0; 5.0) |  |  | 90 | 3.0 (1.0; 5.0) |  |  |
|  | CFB at 3 years | 368 | 0.6 (2.3) | | 39 | 1.2 (2.7) | 0.7 | 0.0; 1.4 | 89 | 1.1 (2.4) | 0.5 | 0.0; 1.0 |
|  | Score at 5 years | 291 | 2.0 (1.0; 4.0) | | 41 | 3.0 (1.0; 5.0) |  |  | 74 | 3.0 (1.0; 5.0) |  |  |
|  | CFB at 5 years | 290 | 0.6 (2.5) | | 41 | 1.1 (3.2) | 0.4 | -0.4; 1.2 | 73 | 1.0 (2.5) | 0.4 | -0.2; 1.0 |
| **Vasomotor**  *Range 0-6* | Baseline score | 404 | 0.0 (0.0; 0.0) | | 40 | 0.0 (0.0; 0.0) |  |  | 107 | 0.0 (0.0; 1.0) |  |  |
|  | Score at 3 months | 385 | 0.0 (0.0; 0.0) | | 37 | 2.0 (2.0; 3.0) |  |  | 104 | 2.0 (0.3; 2.0) |  |  |
|  | CFB at 3 months | 382 | 0.0 (0.7) | | 37 | 2.1 (1.2) | 2.0 | **1.8; 2.3** | 103 | 1.0 (1.1) | 1.1 | **0.9; 1.3** |
|  | Score at 1 year | 386 | 0.0 (0.0; 1.0) | | 42 | 2.0 (2.0; 4.0) |  |  | 100 | 1.0 (0.0; 2.0) |  |  |
|  | CFB at 1 year | 385 | 0.1 (0.8) | | 41 | 2.1 (1.4) | 2.0 | **1.7; 2.3** | 99 | 0.8 (1.1) | 0.8 | **0.6; 1.0** |
|  | Score at 3 years | 370 | 0.0 (0.0; 1.0) | | 40 | 2.0 (1.0; 2.8) |  |  | 90 | 1.0 (0.0; 2.0) |  |  |
|  | CFB at 3 years | 368 | 0.2 (0.9) | | 39 | 1.2 (1.6) | 1.2 | **0.9; 1.6** | 89 | 1.0 (1.2) | 0.7 | **0.5; 1.0** |
|  | Score at 5 years | 291 | 0.0 (0.0; 1.0) | | 41 | 2.0 (1.0; 3.0) |  |  | 74 | 1.0 (0.0; 2.0) |  |  |
|  | CFB at 5 years | 290 | 0.3 (1.0) | | 41 | 1.6 (1.4) | 0.9 | **0.5; 1.3** | 73 | 0.6 (1.4) | 0.4 | **0.1; 0.7** |
| **Sexual**  *Range 0-3* | Baseline score | 404 | 0.0 (0.0; 1.0) | | 40 | 1.0 (0.0; 1.0) |  |  | 107 | 0.0 (0.0; 1.0) |  |  |
|  | Score at 3 months | 385 | 0.0 (0.0; 1.0) | | 37 | 1.0 (0.0; 2.0) |  |  | 104 | 0.5 (0.0; 1.0) |  |  |
|  | CFB at 3 months | 382 | 0.0 (0.7) | | 37 | 0.3 (0.9) | 0.4 | **0.1; 0.6** | 103 | 0.1 (0.9) | 0.1 | -0.1; 0.3 |
|  | Score at 1 year | 386 | 0.0 (0.0; 1.0) | | 42 | 2.0 (1.0; 2.0) |  |  | 100 | 1.0 (0.0; 1.0) |  |  |
|  | CFB at 1 year | 385 | 0.0 (0.7) | | 41 | 0.9 (0.9) | 0.8 | **0.5; 1.0** | 99 | 0.2 (0.9) | 0.3 | **0.1; 0.4** |
|  | Score at 3 years | 370 | 0.0 (0.0; 1.0) | | 40 | 2.0 (1.0; 2.0) |  |  | 90 | 1.0 (0.0; 2.0) |  |  |
|  | CFB at 3 years | 368 | 0.2 (0.9) | | 39 | 0.8 (0.9) | 0.6 | **0.3; 0.9** | 89 | 0.4 (1.0) | 0.3 | **0.1; 0.5** |
|  | Score at 5 years | 291 | 1.0 (0.0; 1.0) | | 41 | 1.0 (1.0; 2.0) |  |  | 74 | 1.0 (0.0; 2.0) |  |  |
|  | CFB at 5 years | 290 | 0.2 (0.8) | | 41 | 0.7 (1.0) | 0.5 | **0.2; 0.8** | 73 | 0.4 (1.0) | 0.3 | **0.1; 0.6** |

The sensitivity analysis excluded participants in the RRS/DO group who had undergone a DO.

Participants were classified into the RRSO group with or without HRT at each time point, based on their HRT use at that time.

The adjusting factors in the analyses are equal to the adjusting factors in the primary analyses of the domains of the Greene Climacteric Scale.

Abbreviations: RRS risk-reducing salpingectomy; DO delayed oophorectomy; RRSO risk-reducing salpingo-oophorectomy; HRT hormone replacement therapy; N number of participants; IQR interquartile range; SD standard deviation; aMD adjusted mean difference; CI confidence interval; CFB change from baseline; ref reference.

| **Table S7. Secondary outcomes** | | | | | | |  |  |  |  |  |  |
| --- | --- | --- | --- | --- | --- | --- | --- | --- | --- | --- | --- | --- |
|  | | **RRS/DO** | | | **RRSO without HRT** | | | | **RRSO with HRT** | | | |
|  |  | N | Median (IQR)  Mean (SD) | *p*-value | N | Median (IQR)  Mean (SD) | aMD | 95% CI | N | Median (IQR)  Mean (SD) | aMD | 95% CI |
| **FSFI** ^a^  *Range 2-36* | Baseline score | 403 | 30.4 (24.6; 32.7) | | 40 | 28.4 (19.3; 31.2) |  |  | 106 | 29.0 (22.9; 31.9) |  |  |
|  | Score at 3 months | 383 | 30.7 (25.4; 33.0) | | 37 | 24.0 (11.9; 30.1) |  |  | 103 | 28.5 (23.6; 31.9) |  |  |
|  | CFB at 3 months | 380 | 0.4 (6.9) | | 37 | -2.3 (9.1) | -3.6 | **-5.8; -1.4** | 101 | 0.1 (7.8) | -0.5 | -2.0; 0.9 |
|  | Score at 1 year | 388 | 30.5 (25.7; 33.0) | | 42 | 21.0 (10.4; 25.4) |  |  | 99 | 28.2 (20.4; 31.7) |  |  |
|  | CFB at 1 year | 387 | 0.4 (7.4) | | 41 | -5.6 (8.6) | -6.2 | **-8.4; -4.1** | 97 | -1.3 (9.9) | -1.9 | **-3.5; -0.3** |
|  | Score at 3 years | 380 | 29.9 (23.5; 32.6) | | 40 | 16.3 (6.1; 28.5) |  |  | 89 | 27.6 (21.2; 31.8) |  |  |
|  | CFB at 3 years | 378 | -0.8 (8.9) | | 38 | -5.4 (8.0) | -5.3 | **-7.9; -2.7** | 88 | -1.3 (10.0) | -0.9 | -2.7; 1.0 |
|  | Score at 5 years | 362 | 28.7 (21.0; 32.3) | | 40 | 21.2 (10.7; 27.1) |  |  | 74 | 25.8 (15.7; 31.4) |  |  |
|  | CFB at 5 years | 360 | -1.6 (9.5) | | 40 | -4.6 (7.1) | -3.1 | **-5.9; -0.3** | 72 | -1.8 (10.4) | -0.6 | -2.8; 1.6 |
| **FSDS** ^b^  *Range 0-48* | Baseline score | 403 | 4.0 (0.0; 11.0) | | 40 | 5.5 (0.0; 20.3) |  |  | 107 | 5.0 (0.0; 13.0) |  |  |
|  | Score at 3 months | 384 | 4.0 (0.0; 12.0) | | 37 | 13.0 (3.5; 24.0) |  |  | 104 | 4.5 (0.0; 17.0) |  |  |
|  | CFB at 3 months | 381 | 0.6 (6.7) | | 37 | 4.1 (9.2) | 5.1 | **2.7; 7.5** | 103 | 0.6 (9.8) | 0.2 | -1.5; 2.0 |
|  | Score at 1 year | 388 | 4.0 (0.0; 12.0) | | 42 | 19.5 (11.3; 27.3) |  |  | 100 | 7.0 (1.0; 16.0) |  |  |
|  | CFB at 1 year | 387 | 0.3 (6.9) | | 41 | 8.5 (10.5) | 8.3 | **5.8; 10.7** | 99 | 1.2 (9.9) | 1.0 | -0.9; 2.9 |
|  | Score at 3 years | 380 | 4.0 (0.0; 13.0) | | 40 | 21.0 (7.3; 28.3) |  |  | 90 | 5.5 (1.0; 14.0) |  |  |
|  | CFB at 3 years | 378 | 1.1 (8.4) | | 39 | 5.8 (11.2) | 5.8 | **2.9; 8.7** | 89 | 1.9 (9.1) | 0.8 | -1.3; 2.9 |
|  | Score at 5 years | 362 | 6.0 (0.0; 14.0) | | 41 | 13.0 (3.5; 23.0) |  |  | 74 | 8.0 (1.0; 18.3) |  |  |
|  | CFB at 5 years | 361 | 2.4 (8.6) | | 41 | 4.6 (11.2) | 4.2 | **1.0; 7.5** | 73 | 2.3 (10.6) | 0.9 | -1.5; 3.2 |
| **CWS** ^c^  *Range 8-32* | Baseline score | 404 | 14.0 (12.0; 17.0) | | 40 | 14.5 (12.0; 18.0) |  |  | 107 | 14.0 (12.0; 17.0) |  |  |
|  | Score at 3 months | 385 | 12.0 (10.0; 15.0) | | 37 | 13.0 (10.0; 16.0) |  |  | 104 | 12.5 (10.0; 15.0) |  |  |
|  | CFB at 3 months | 382 | -1.5 (2.7) | | 37 | -1.6 (3.8) | -0.2 | -1.0; 0.7 | 103 | -1.8 (3.3) | -0.4 | -1.0; 0.2 |
|  | Score at 1 year | 389 | 12.0 (10.0; 15.0) | | 42 | 13.0 (10.0; 16.0) |  |  | 100 | 12.0 (9.0; 14.0) |  |  |
|  | CFB at 1 year | 388 | -1.6 (3.0) | | 41 | -1.6 (4.1) | 0.0 | -0.9; 0.9 | 99 | -2.3 (3.7) | -0.7 | -1.3; 0.0 |
|  | Score at 3 years | 382 | 13.0 (10.0; 15.0) | | 40 | 12.0 (10.0; 14.0) |  |  | 90 | 11.0 (9.0; 13.0) |  |  |
|  | CFB at 3 years | 380 | -1.6 (3.3) | | 39 | -2.3 (4.2) | -0.6 | -1.6; 0.3 | 89 | -2.7 (3.4) | -1.2 | **-1.9; -0.6** |
|  | Score at 5 years | 363 | 12.0 (10.0; 14.0) | | 41 | 12.0 (10.5; 14.0) |  |  | 74 | 11.0 (9.0; 13.0) |  |  |
|  | CFB at 5 years | 362 | -1.7 (3.3) | | 41 | -2.1 (4.8) | -1.0 | **-2.0; -0.1** | 73 | -2.7 (3.9) | -1.3 | **-1.9; -0.5** |
| **DRS** ^d^  *Range 0-100* | Score at 1 year | 383 | 5.0 (0.0; 25.0) | | 41 | 10.0 (2.5; 27.5) |  |  | 94 | 5.0 (0.0; 20.0) |  |  |
|  | Score at 5 years | 355 | 5.0 (0.0; 20.0) | | 40 | 12.5 (5.0; 28.5) |  |  | 73 | 10.0 (0.0; 22.5) |  |  |
| **SF-36 PCS** ^e^  *Range 0-100* | Baseline score | 405 | 56.7 (52.7; 59.0) | | 40 | 49.7 (41.6; 56.8) |  |  | 107 | 56.9 (54.0; 59.2) |  |  |
|  | Score at 3 months | 385 | 56.7 (52.3; 59.0) | | 37 | 53.5 (46.2; 57.5) |  |  | 104 | 55.9 (48.2; 58.1) |  |  |
|  | CFB at 3 months | 382 | 0.0 (6.3) | | 37 | 1.7 (6.6) | -1.2 | -3.4; 1.1 | 103 | -2.1 (7.2) | -1.7 | **-3.2; -0.3** |
|  | Score at 1 year | 389 | 56.9 (52.3; 59.1) | | 42 | 53.2 (46.9; 57.3) |  |  | 100 | 57.0 (50.5; 59.1) |  |  |
|  | CFB at 1 year | 388 | 0.1 (6.7) | | 41 | -0.1 (10.7) | -4.1 | **-6.4; -1.8** | 99 | -0.5 (7.4) | -0.9 | -2.5; 0.7 |
|  | Score at 3 years | 382 | 56.6 (52.3; 58.8) | | 40 | 55.3 (49.4; 58.2) |  |  | 90 | 55.5 (51.7; 59.0) |  |  |
|  | CFB at 3 years | 380 | -0.4 (7.2) | | 39 | 2.2 (7.5) | 0.6 | -1.9; 3.2 | 89 | -0.9 (6.2) | -0.1 | -1.8; 1.6 |
|  | Score at 5 years | 360 | 56.2 (50.3; 58.6) | | 41 | 53.7 (46.4; 57.5) |  |  | 74 | 55.1 (49.9; 58.4) |  |  |
|  | CFB at 5 years | 359 | -0.7 (7.3) | | 41 | 1.1 (7.8) | 1.5 | -1.1; 4.0 | 73 | -1.3 (7.0) | -0.4 | -2.2; 1.4 |
| **SF-36 MCS** ^f^  *Range 0-100* | Baseline score | 405 | 54.0 (47.7; 57.3) | | 40 | 47.9 (41.6; 55.0) |  |  | 107 | 54.0 (48.4; 57.2) |  |  |
|  | Score at 3 months | 385 | 54.1 (47.9; 57.5) | | 37 | 47.2 (39.5; 52.9) |  |  | 104 | 53.6 (43.5; 56.4) |  |  |
|  | CFB at 3 months | 382 | -0.3 (7.6) | | 37 | -0.6 (9.2) | -1.5 | -4.1; 1.1 | 103 | -1.4 (9.4) | -0.4 | -2.0; 1.3 |
|  | Score at 1 year | 389 | 54.1 (48.1; 57.2) | | 42 | 50.8 (41.7; 55.8) |  |  | 100 | 53.0 (43.2; 56.5) |  |  |
|  | CFB at 1 year | 388 | -0.5 (9.0) | | 41 | 1.0 (11.6) | -0.7 | -3.4; 2.1 | 99 | -2.3 (9.7) | -1.0 | -2.8; 0.9 |
|  | Score at 3 years | 382 | 54.2 (48.9; 57.4) | | 40 | 52.8 (45.8; 56.6) |  |  | 90 | 53.2 (45.0; 56.6) |  |  |
|  | CFB at 3 years | 380 | 0.1 (9.4) | | 39 | 3.3 (10.4) | 1.4 | -1.4; 4.1 | 89 | -1.4 (10.6) | -0.8 | -2.6; 1.1 |
|  | Score at 5 years | 360 | 53.6 (46.5; 56.6) | | 41 | 53.3 (46.5; 57.0) |  |  | 74 | 53.5 (45.2; 56.7) |  |  |
|  | CFB at 5 years | 359 | -1.1 (9.4) | | 41 | 3.7 (11.4) | 3.1 | **0.4; 5.7** | 73 | -1.2 (12.0) | 0.5 | -1.6; 2.6 |

Participants were classified into the RRSO group with or without HRT at each time point, based on their HRT use at that time.

Abbreviations: RRS risk-reducing salpingectomy; DO delayed oophorectomy; RRSO risk-reducing salpingo-oophorectomy; HRT hormone replacement therapy; N number of participants; IQR interquartile range; SD standard deviation; aMD adjusted mean difference; CI confidence interval; CFB change from baseline; ref reference; FSFI Female Sexual Functioning Index; FSDS Female Sexual Distress Scale; CWS Cancer Worry Scale; DRS Decision Regret Scale; SF-36 Short Form Health Survey 36; PCS physical component score; MCS mental component score.

^a^ Adjusting factors in the analysis: type of strategy, type of *BRCA*-PV, follow-up time point, age at first surgery, baseline GCS score, baseline FSFI score, a random effect for centre of inclusion, an unstructured correlation matrix for the within-participants correlation between repeated measurements, and a two-way interaction for type of strategy by follow-up time point.

^b^ Adjusting factors in the analysis: type of strategy, type of *BRCA*-PV, follow-up time point, age at first surgery, baseline FSDS score, a random effect for centre of inclusion, an unstructured correlation matrix for the within-participants correlation between repeated measurements, a three-way interaction for type of strategy by follow-up time point by age at first surgery, and its corresponding two-way interactions.

^c^ Adjusting factors in the analysis: type of strategy, type of *BRCA*-PV, follow-up time point, age at first surgery, baseline GCS score, baseline CWS score, baseline SF-36 physical component score, a random effect for centre of inclusion, an unstructured correlation matrix for the within-participants correlation between repeated measurements, a three-way interaction for type of strategy by follow-up time point by baseline CWS score, and its corresponding two-way interactions.

^d^ Adjusting factors in the analysis: type of strategy, type of *BRCA*-PV, age at first surgery, a random effect for centre of inclusion.

^e^ Adjusting factors in the analysis: type of strategy, type of *BRCA*-PV, follow-up time point, age at first surgery, baseline GCS score, baseline SF-36 physical component score, a random effect for centre of inclusion, an unstructured correlation matrix for the within-participants correlation between repeated measurements, a three-way interaction for type of strategy by follow-up time point by baseline SF-36 physical component score, a three-way interaction for type of strategy by follow-up time point by age at first surgery, and its corresponding two-way interactions.

^f^ Adjusting factors in the analysis: type of strategy, type of *BRCA*-PV, follow-up time point, age at first surgery, baseline GCS score, baseline SF-36 physical component score, baseline SF-36 mental component score, a random effect for centre of inclusion, an unstructured correlation matrix for the within-participants correlation between repeated measurements, a three-way interaction for type of strategy by follow-up time point by baseline SF-36 mental component score, and its corresponding two-way interactions.

Proportion of participants with sexual dysfunction

|  | Pre-surgery | 3 months | 1 year | 3 years | 5 years |
| --- | --- | --- | --- | --- | --- |
| RRS/DO | 30.5% | 27.7% | 27.3% | 34.7% | 38.2% |
| RRSO without HRT | 37.5% | 62.2% | 81% | 72.5% | 72.5% |
| RRSO with HRT | 34.9% | 36.9% | 44.4% | 46.1% | 54.1% |

**Figure S2. Proportions of participants with sexual dysfunction per time period**

Sexual dysfunction was indicated by a score of 26.55 or lower on the Female Sexual Functioning Index (FSFI). Abbreviations: DO: delayed oophorectomy; HRT: hormonal replacement therapy; RRS: risk-reducing salpingectomy; RRSO: risk-reducing salpingo-oophorectomy

Proportion of participants with sexual distress

|  | Pre-surgery | 3 months | 1 year | 3 years | 5 years |
| --- | --- | --- | --- | --- | --- |
| RRS/DO | 17.6% | 18.8% | 17.5% | 22.1% | 24.8% |
| RRSO without HRT | 30% | 48.6% | 71.4% | 62.5% | 47.4% |
| RRSO with HRT | 19.6% | 27.9% | 27% | 23.3% | 38.4% |

**Figure S3. Proportions of participants with sexual distress per time period**

Relevant sexual distress was indicated by a score of 15 or higher on the Female Sexual Distress Scale (FSDS). Abbreviations: DO: delayed oophorectomy; HRT: hormonal replacement therapy; RRS: risk-reducing salpingectomy; RRSO: risk-reducing salpingo-oophorectomy

Proportion of participants with high levels of cancer worry

|  | Pre-surgery | 3 months | 1 year | 3 years | 5 years |
| --- | --- | --- | --- | --- | --- |
| RRS/DO | 55.7% | 36.4% | 36.2% | 36.6% | 34.4% |
| RRSO without HRT | 57.5% | 45.9% | 47.6% | 30% | 26.8% |
| RRSO with HRT | 59.8% | 37.5% | 32% | 21.1% | 23% |

**Figure S4. Proportions of participants with high cancer worry per time period**

High levels of cancer worry were indicated by a score of 14 or higher on the Cancer Worry Scale (CWS). Abbreviations: DO: delayed oophorectomy; HRT: hormonal replacement therapy; RRS: risk-reducing salpingectomy; RRSO: risk-reducing salpingo-oophorectomy

| **Table S8. Sensitivity analysis excluding women with both RRS and DO at the moment of follow-up of the secondary outcomes** | | | | | | | | | | | | |
| --- | --- | --- | --- | --- | --- | --- | --- | --- | --- | --- | --- | --- |
|  | | **RRS without DO** | | | **RRSO without HRT** | | | | **RRSO with HRT** | | | |
|  |  | N | Median (IQR)  Mean (SD) | *p*-value | N | Median (IQR)  Mean (SD) | aMD | 95% CI | N | Median (IQR)  Mean (SD) | aMD | 95% CI |
| **FSFI**  *Range 2-36* | Baseline score | 403 | 30.4 (24.6; 32.7) | | 40 | 28.4 (19.3; 31.2) |  |  | 106 | 29.0 (22.9; 31.9) |  |  |
|  | Score at 3 months | 383 | 30.7 (25.4; 33.0) | | 37 | 24.0 (11.9; 30.1) |  |  | 103 | 28.5 (23.6; 31.9) |  |  |
|  | CFB at 3 months | 380 | 0.4 (6.9) | | 37 | -2.3 (9.1) | -3.7 | **-5.9; -1.5** | 101 | 0.1 (7.8) | -0.6 | -2.1; 0.9 |
|  | Score at 1 year | 385 | 30.5 (25.7; 33.0) | | 42 | 21.0 (10.4; 25.4) |  |  | 99 | 28.2 (20.4; 31.7) |  |  |
|  | CFB at 1 year | 384 | 0.5 (7.3) | | 41 | -5.6 (8.6) | -6.3 | **-8.4; -4.2** | 97 | -1.3 (9.9) | -2.0 | **-3.6; -0.4** |
|  | Score at 3 years | 369 | 30.0 (23.8; 32.6) | | 40 | 16.3 (6.1; 28.5) |  |  | 89 | 27.6 (21.2; 31.8) |  |  |
|  | CFB at 3 years | 367 | -0.7 (8.9) | | 38 | -5.4 (8.0) | -5.4 | **-8.0; -2.9** | 88 | -1.3 (10.0) | -1.0 | -2.9; 0.9 |
|  | Score at 5 years | 289 | 29.2 (23.5; 32.4) | | 40 | 21.2 (10.7; 27.1) |  |  | 74 | 25.8 (15.7; 31.4) |  |  |
|  | CFB at 5 years | 288 | -1.0 (9.4) | | 40 | -4.6 (7.1) | -4.0 | **-6.7; -1.3** | 72 | -1.8 (10.4) | -1.5 | -3.6; 0.7 |
| **FSDS**  *Range 0-48* | Baseline score | 403 | 4.0 (0.0; 11.0) | | 40 | 5.5 (0.0; 20.3) |  |  | 107 | 5.0 (0.0; 13.0) |  |  |
|  | Score at 3 months | 384 | 4.0 (0.0; 12.0) | | 37 | 13.0 (3.5; 24.0) |  |  | 104 | 4.5 (0.0; 17.0) |  |  |
|  | CFB at 3 months | 381 | 0.6 (6.7) | | 37 | 4.1 (9.2) | 5.1 | **2.7; 7.6** | 103 | 0.6 (9.8) | 0.3 | -1.5; 2.0 |
|  | Score at 1 year | 385 | 4.0 (0.0; 12.0) | | 42 | 19.5 (11.3; 27.3) |  |  | 100 | 7.0 (1.0; 16.0) |  |  |
|  | CFB at 1 year | 384 | 0.3 (7.0) | | 41 | 8.5 (10.5) | 8.2 | **5.8; 10.7** | 99 | 1.2 (9.9) | 1.0 | -0.9; 2.9 |
|  | Score at 3 years | 369 | 4.0 (0.0; 13.0) | | 40 | 21.0 (7.3; 28.3) |  |  | 90 | 5.5 (1.0; 14.0) |  |  |
|  | CFB at 3 years | 367 | 1.1 (8.3) | | 39 | 5.8 (11.2) | 5.8 | **3.0; 8.7** | 89 | 1.9 (9.1) | 0.9 | -1.2; 3.0 |
|  | Score at 5 years | 290 | 6.0 (0.0; 14.0) | | 41 | 13.0 (3.5; 23.0) |  |  | 74 | 8.0 (1.0; 18.3) |  |  |
|  | CFB at 5 years | 289 | 1.8 (8.1) | | 41 | 4.6 (11.2) | 4.7 | **1.6; 7.8** | 73 | 2.3 (10.6) | 1.4 | -0.9; 3.6 |
| **CWS**  *Range 8-32* | Baseline score | 404 | 14.0 (12.0; 17.0) | | 40 | 14.5 (12.0; 18.0) |  |  | 107 | 14.0 (12.0; 17.0) |  |  |
|  | Score at 3 months | 385 | 12.0 (10.0; 15.0) | | 37 | 13.0 (10.0; 16.0) |  |  | 104 | 12.5 (10.0; 15.0) |  |  |
|  | CFB at 3 months | 382 | -1.5 (2.7) | | 37 | -1.6 (3.8) | -0.2 | -1.1; 0.6 | 103 | -1.8 (3.3) | -0.4 | -1.0; 0.1 |
|  | Score at 1 year | 386 | 12.0 (10.0; 15.0) | | 42 | 13.0 (10.0; 16.0) |  |  | 100 | 12.0 (9.0; 14.0) |  |  |
|  | CFB at 1 year | 385 | -1.6 (3.0) | | 41 | -1.6 (4.1) | 0.0 | -1.0; 0.9 | 99 | -2.3 (3.7) | -0.7 | **-1.4; -0.1** |
|  | Score at 3 years | 370 | 12.5 (10.0; 15.0) | | 40 | 12.0 (10.0; 14.0) |  |  | 90 | 11.0 (9.0; 13.0) |  |  |
|  | CFB at 3 years | 368 | -1.6 (3.3) | | 39 | -2.3 (4.2) | -0.7 | -1.6; 0.3 | 89 | -2.7 (3.4) | -1.3 | **-1.9; -0.6** |
|  | Score at 5 years | 290 | 12.0 (10.0; 14.0) | | 41 | 12.0 (10.5; 14.0) |  |  | 74 | 11.0 (9.0; 13.0) |  |  |
|  | CFB at 5 years | 289 | -1.6 (3.4) | | 41 | -2.1 (4.8) | -1.2 | **-2.2; -0.2** | 73 | -2.7 (3.9) | -1.4 | **-2.1; -0.6** |
| **DRS**  *Range 0-100* | Score at 1 year | 382 | 5.0 (0.0; 25.0) | | 41 | 10.0 (2.5; 27.5) |  |  | 94 | 5.0 (0.0; 20.0) |  |  |
|  | Score at 5 years | 288 | 5.0 (0.0; 20.0) | | 40 | 12.5 (5.0; 28.5) |  |  | 73 | 10.0 (0.0; 22.5) |  |  |
| **SF-36 PCS**  *Range 0-100* | Baseline score | 405 | 56.7 (52.7; 59.0) | | 40 | 49.7 (41.6; 56.8) |  |  | 107 | 56.9 (54.0; 59.2) |  |  |
|  | Score at 3 months | 385 | 56.7 (52.3; 59.0) | | 37 | 53.5 (46.2; 57.5) |  |  | 104 | 55.9 (48.2; 58.1) |  |  |
|  | CFB at 3 months | 382 | 0.0 (6.3) | | 37 | 1.7 (6.6) | -1.2 | -3.5; 1.1 | 103 | -2.1 (7.2) | -1.7 | **-3.2; -0.3** |
|  | Score at 1 year | 386 | 56.9 (52.4; 59.2) | | 42 | 53.2 (46.9; 57.3) |  |  | 100 | 57.0 (50.5; 59.1) |  |  |
|  | CFB at 1 year | 385 | 0.0 (6.7) | | 41 | -0.1 (10.7) | -4.2 | **-6.5; -1.8** | 99 | -0.5 (7.4) | -0.9 | -2.5; 0.7 |
|  | Score at 3 years | 370 | 56.7 (52.4; 58.9) | | 40 | 55.3 (49.4; 58.2) |  |  | 90 | 55.5 (51.7; 59.0) |  |  |
|  | CFB at 3 years | 368 | -0.5 (7.3) | | 39 | 2.2 (7.5) | 0.6 | -2.0; 3.2 | 89 | -0.9 (6.2) | -0.1 | -1.8; 1.6 |
|  | Score at 5 years | 291 | 56.7 (52.3; 59.0) | | 41 | 53.7 (46.4; 57.5) |  |  | 74 | 55.1 (49.9; 58.4) |  |  |
|  | CFB at 5 years | 290 | -0.3 (6.7) | | 41 | 1.1 (7.8) | 1.2 | -1.2; 3.6 | 73 | -1.3 (7.0) | -0.7 | -2.4; 1.0 |
| **SF-36 MCS**  *Range 0-100* | Baseline score | 405 | 54.0 (47.7; 57.3) | | 40 | 47.9 (41.6; 55.0) |  |  | 107 | 54.0 (48.4; 57.2) |  |  |
|  | Score at 3 months | 385 | 54.1 (47.9; 57.5) | | 37 | 47.2 (39.5; 52.9) |  |  | 104 | 53.6 (43.5; 56.4) |  |  |
|  | CFB at 3 months | 382 | -0.3 (7.6) | | 37 | -0.6 (9.2) | -1.6 | -4.3; 1.0 | 103 | -1.4 (9.4) | -0.4 | -2.1; 1.2 |
|  | Score at 1 year | 386 | 54.1 (47.9; 57.2) | | 42 | 50.8 (41.7; 55.8) |  |  | 100 | 53.0 (43.2; 56.5) |  |  |
|  | CFB at 1 year | 385 | -0.5 (9.0) | | 41 | 1.0 (11.6) | -0.8 | -3.5; 2.0 | 99 | -2.3 (9.7) | -1.0 | -2.9; 0.9 |
|  | Score at 3 years | 370 | 54.2 (49.0; 57.5) | | 40 | 52.8 (45.8; 56.6) |  |  | 90 | 53.2 (45.0; 56.6) |  |  |
|  | CFB at 3 years | 368 | 0.2 (9.3) | | 39 | 3.3 (10.4) | 1.1 | -1.6; 3.8 | 89 | -1.4 (10.6) | -1.0 | -2.8; 0.9 |
|  | Score at 5 years | 291 | 54.0 (47.7; 56.7) | | 41 | 53.3 (46.5; 57.0) |  |  | 74 | 53.5 (45.2; 56.7) |  |  |
|  | CFB at 5 years | 290 | -0.5 (9.1) | | 41 | 3.7 (11.4) | 2.5 | -0.1; 5.0 | 73 | -1.2 (12.0) | -0.1 | -2.2; 1.9 |

Participants were classified into the RRSO group with or without HRT at each time point, based on their HRT use at that time. The adjusting factors in the analyses are equal to the adjusting factors in the primary analyses of the secondary outcomes.

Abbreviations: RRS risk-reducing salpingectomy; DO delayed oophorectomy; RRSO risk-reducing salpingo-oophorectomy; HRT hormone replacement therapy; N number of participants; IQR interquartile range; SD standard deviation; aMD adjusted mean difference; CI confidence interval; CFB change from baseline; ref reference; FSFI Female Sexual Functioning Index; FSDS Female Sexual Distress Scale; CWS Cancer Worry Scale; DRS Decision Regret Scale; SF-36 Short Form Health Survey 36; PCS physical component score; MCS mental component score.
